# Multidisciplinary optimization of automotive mega-castings merging classical structural optimization with response-surface-based optimization enhanced by machine learning

**DOI:** 10.1038/s41598-023-47937-5

**Published:** 2023-12-07

**Authors:** Jens Triller, Marta L. Lopez, Matthias Nossek, Moritz A. Frenzel

**Affiliations:** 1 ALTAIR Engineering GmbH, Calwer Str. 7, 71034 Böblingen, Germany; 2 BMW Group, Knorrstraße 147, 80788 Munich, Germany

**Keywords:** Mechanical engineering, Computational science

## Abstract

Large high pressure die castings (HPDC), recently referred to as mega-castings, can replace plenty of steel metal sheets usually employed for body-in-white (BIW) structures. They can save manufacturing expense and unleash additional lightweight potential thanks to additional design freedom and material properties. The BIW plays a major role in automotive design since it must fulfill numerous structural targets ranging from stiffness for vehicle dynamics, dynamic responses for NVH (noise, vibration, harshness), driving comfort standards and several passive safety requirements. The use of mega-casting structures leads to additional requirements with respect to castability and material quality. Achieving a lightweight design considering requirements related to crash or castability is a challenge on its own, due to the high computational cost of related simulation techniques. Considering multiple requirements simultaneously, therefore often leads to non-weight-optimal structures. To exploit the full lightweight potential, we present a generative multidisciplinary optimization pipeline for the structural design of automotive mega-casting parts in this paper. The approach combines established methods in automotive industry such as topology optimization and response-surface-based (RSM) optimization and enhances the latter by machine learning (ML) based clustering and classification. In a first step topology optimization is employed to derive optimal load-paths for multidisciplinary loading conditions. For this purpose, casting manufacturing constraints as well as more than hundred linearized loads are used to incorporate NVH and passive safety requirements. In a next step the optimal thickness distribution and rib orientation of the structure is achieved using RSM optimization algorithms for the computationally expensive nonlinear crash and casting simulations. Performance indicators are treated by unsupervised learning based on clustering. This enables classification constraints based on simulation field results from hundreds of samples to be included into RSM optimization. It resolves a typical risk of pure scalar, regression-type targets, where supposed optimal results fail when domain experts examine the full field result of the corresponding simulation. It is shown how this approach is superior in achieving a weight-optimal design and turnaround time compared to a design workflow classically used for BIW structures.

## Introduction

The shift towards battery electric vehicles as the new norm, driven by the imperative to reduce harmful emissions, increases the pressure on Original Equipment Manufacturers (OEMs) to reduce time and cost^1–3^. While the pursuit of weight reduction remains ongoing, the inclusion of batteries introduces additional weight. Multiple OEMs have focused on advanced manufacturing processes, including so-called “mega-casting” for BIW structures^1–3^, as they provide engineers more design freedom in comparison with the standard stamped steel plate designs and unleash additional lightweighting potential. Molten metal alloy is forced into a reusable mold under high pressure and speed. After solidification the mold is opened, and the finished piece is removed. This represents a small amount of manufacturing steps in comparison to stamping steel plates, positioning, and welding. Industrial examples have shown that mega-castings can significantly reduce the number of parts assembled in BIW structures^4^ and therefore can save manufacturing time and cost^3^. Moreover, this supports achieving circular economy through usage of recycled aluminum.

The previously outlined benefits come with a list of challenges to be solved. The basic prerequisite to produce such BIW parts is the availability of correspondingly large HPDC machines. Although, the number of toolmaker capable of producing such machinery is very limited, there have been significant improvements in recent years^5^. Locking forces up to 9000 tons can be reached, enabling high injection speeds and the manufacturing of large casting parts^5^. Another hurdle to be taken is maintaining tight manufacturing tolerances, which is addressed in Ref.^6^. In addition, the increased size and complexity of mega-castings hampers achieving sufficient casting quality. In the design of such components not only traditional mechanical requirements but also casting process and material quality play an important role. Uniformly filling the casting-component and short filling times are crucial in that regard. Achieving sufficient material quality is especially important to maintain the BIW’s crashworthiness, as aluminum tends to be brittle in contrast to the conventional BIW made of steel. Tesla has addressed this issue in a patent recently^7^. However, according to Ref.^5^ this design contradicts the lightweighting target.

In addition, there are many other requirements not directly related to the HPDC to be met. The BIW contributes significantly to the fulfilment of criteria related, e.g. to static stiffness for vehicle dynamics, NVH, driving comfort standards and passive safety. While for the evaluation of the first efficient linear statics FE-solvers are at hand, the latter requires extensive use of High-Performance Computing to simulate several destructive crash tests.

**Figure 1:**
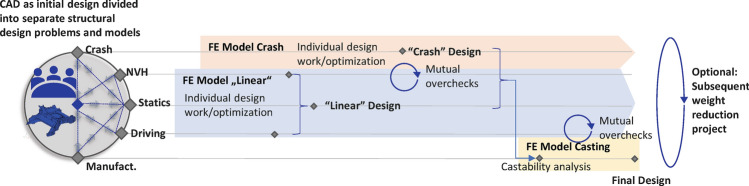
Classical design approach frequently used for the design of BIW structures.

To develop a structure that attends above, multiple requirements from different disciplines and multiple processes must run in parallel. Each discipline, e.g., NVH, statics, durability and crash develop their own FE-Models and run different calculations. It is important to notice that due to different commercial solvers and physics involved in each discipline, different mesh types and model sizes may be required. As a result, each department in large OEMs works with an independent model. Often structural optimization techniques are employed to derive structural changes improving their discipline targets. But due to missing alignment of separate design swim lanes non-optimal solutions with respect to structural synergy and weight potential are derived. Consequently, a synchronization phase is needed in which all design changes are combined. Manufacturing analyses typically take the resulting structural designs as input and mutual overchecking is only performed on critical hot-spot locations. Such process is illustrated in Fig. 1 and will be referred to in the following as classical design approach.

The process described is laborious and presents multiple challenges, especially for large castings, since the combination of the design directions provided by disciplines separately does not necessarily lead to a resulting lightweight structure that fulfills all targets. Thus, the process requires several iterations among multiple departments. This contradicts the stated goal of reducing development time and cost.

**Figure 2:**
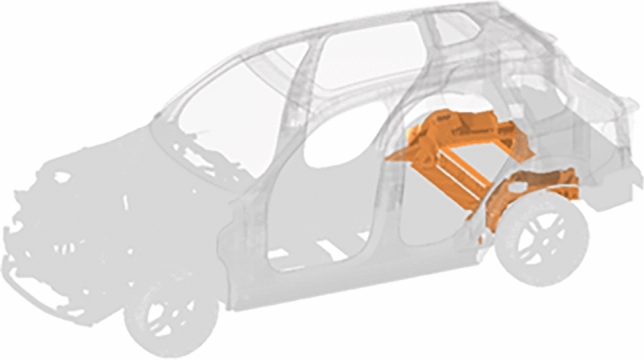
Large casting component (orange) in BIW structure.

In this work, a large casting component, illustrated in Fig. 2, is used as case study. The component represents large portion of the rear of the BIW, and its structural performance must cover diverse NVH requirements, crash, stiffness and driving conditions.

This paper targets on providing a strategy how to avoid design iteration by employing multidisciplinary design optimization (MDO) within a generative design pipeline. MDO for the design of BIW structures has been addressed by several studies^8–11^. For comprehensive review on MDO, refer for instance to Cramer et al.^12^, Sobieszczanski-Sobieski and Haftka^13^, and most recently Martins and Lambe^14^.

Key aspect of MDO is the coupling of single discipline problems. A common example of a strongly coupled physical problem is aeroelasticity. In optimization the coupling often times is determined by common design variables, e.g. beam sections or thicknesses of structural parts, where individual physical phenomena such as structural stiffness or drag can be analyzed sufficiently exact separately.

In the case of the industrial design problem of mega-casting parts within a BIW structure the coupling design variables for the MDO can be summarized as follows: the geometry including base surface with varying thickness, rib positions, orientations and thicknesses and draw directions, eventually separated in different sections.

As design disciplines we define the various structural requirements arising from the full car load conditions, comprised by static stiffness, driving dynamics, NVH (noise vibration harshness) and crash requirements. While these can all be summarized by the physical discipline of structural mechanics, they separate into different disciplines when analyzed in detail. In the dynamic regime for instance the considered frequency spectrum plays an important role, whereas for crash loading not only stiffness but also energy absorption is key to fulfill passive safety requirements. This implies application of separate computational solver techniques ranging from implicit to explicit finite element solvers and varying modelling techniques and mesh resolutions. We demonstrate in the remaining chapters how we handle the various structural disciplines coupled by the common design space of the casting part geometry.

Referring to physical fields as design disciplines the industrial problem of mega-casting design imposes additionally the filling of the die as computational fluid dynamics problem. Although, there is no physical interaction between the die filling process and the structural loading of the final assembled part, the coupling design variables describing the geometry of the part play a significant role. Die filling of such large parts with sufficient material quality is challenging, particularly for high material quality necessary to achieve ductility required for crash. Thickness, orientation, and positioning of ribs define the material flow of the die filling and therefore constrain the optimal design for structural requirements. This additional physical field of material flow makes multidisciplinary optimization inevitable to design mega-casting structures.

To resolve such a MDO problem to a great extend using optimization algorithms we apply two different architectures within a sequential process. In the first phase we seek design proposals that capture stiffness load-paths for all disciplines while respecting manufacturing constraints from casting process using topology and free-size optimization. To enable handling a huge number of design variables we transform the structural multi-discipline requirements into a purely static multi-load problem by linearization and integrated casting constraints. Requirements in the dynamic regime or stemming from energy absorption in crash are transformed to pure stiffness requirements in several load cases. Balancing these load cases represents balancing of the different structural disciplines but offers application of one single optimization solver. The balancing process itself results in several different design proposals, where the best candidate design can be selected afterwards.

In the second phase, when the part design has already evolved, we overcome the simplifying load linearization and include nonlinear structural simulations and the die filling problem of the casting process. Thus, we apply a multidisciplinary feasible architecture^12^ evaluating response functions from structural analyses and die filling simulations. To leverage full insight of each individual nonlinear simulation we implement a novel technique based on machine learning algorithms to cluster and classify simulation results. By doing so, a single discipline expert's evaluation of simulation field result, can be scaled to hundreds of simulation results and algorithmically implemented into the multidisciplinary optimization process.

**Figure 3:**
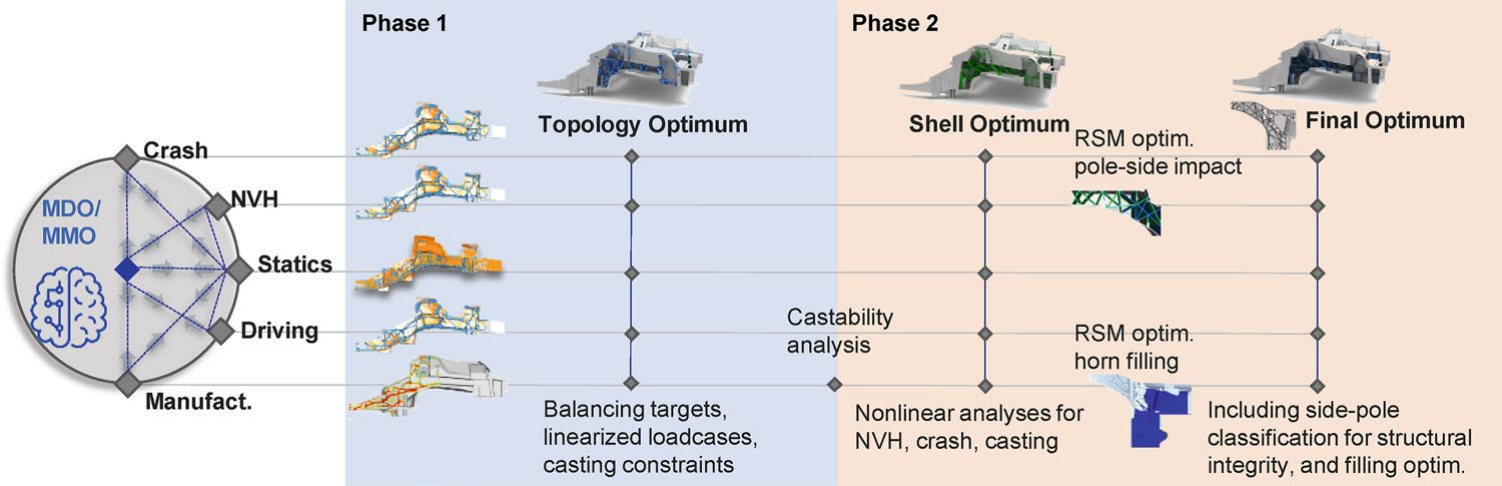
Generative design process highlighting two phases and separate disciplines.

The two-phase generative design process, as illustrated in Fig. 3, will be demonstrated in the following using the depicted large casting component as real industry application example.

The sequence of the individual process steps is reflected by the structure of the paper. Chapter 2 showcases the results of phase-one, while simultaneously providing theoretical background to the optimized load cases as well as optimization techniques. Afterwards RSM-based optimization is introduced in chapter 3. In Chapter 4 and 5 the RSM-based optimization of the pole-side impact and the casting process are shown and novel enhancements like clustering and classification are introduced. The final resulting structure is validated through a comparison with a design stemming from the classical design approach as illustrated in Fig. 1. Finally, a summary and conclusion is worked out.

## Optimal load-path conceptual design of casting structure including multidisciplinary load requirements like crash

**Figure 4:**
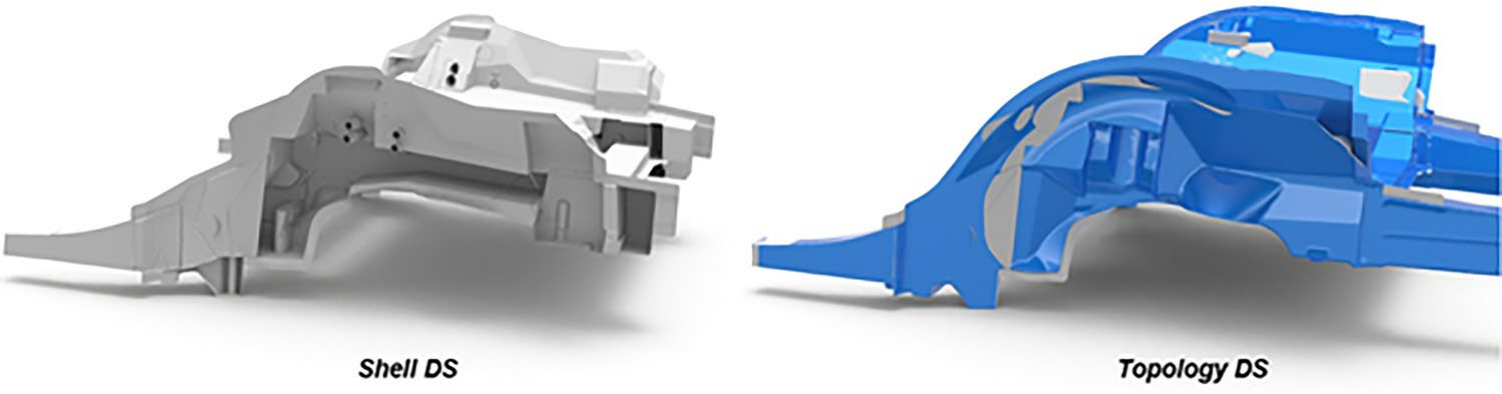
Casting component—design space and base-shell structure.

The first phase of the generative design approach (Fig. 3) focuses on deriving a load-path conceptual design. As illustrated in Fig. 4, the available design space (DS) can be split into the shell DS specified by base surfaces connected to the remaining parts of the BIW and the topology DS.

Each DS relates to a different optimization technique. The shell DS is optimized using free-size optimization, in which the thickness of each shell element is varied while the topology DS undergoes density-based topology optimization. The concept of topology optimization is tremendously powerful in mechanical engineering, since it provides answers for important design questions, such as “where to place material most efficiently?”.

Topology optimization concept was firstly introduced in 1988 by Bendsøe and Kikuchi^15^ and it has developed into many directions. A comprehensible review of more recent approaches for topology optimization can be found at Ref.^16^.

In this work, the topology optimization problem is tackled by using commercial tools (*OptiStruct*)^17^ employing the Solid Isotropic Material Penalization (SIMP) approach. Here, the element densities are varied and related to their mechanical properties, i.e., their Youngs modulus. Multiple works have addressed this approach and are documented in Refs.^18–20^.

As the number of elements exceeds 6 million here, the optimizer must be able to handle a huge number of design variables ${n}_{\mathrm{d}}$. For linear static response optimization problems, the sensitivities regarding the design variables $\mathbf{x}$ can be calculated very efficiently even for a huge number of design variables employing adjoint sensitivity analysis^21^. Speaking of linear static response optimization means that the responses involved in the optimization result of solving the FE-equation of linear statics:

1 $$\mathbf{K}(\mathbf{x})\mathbf{u}=\mathbf{f}$$

with the stiffness matrix $\mathbf{K}(\mathbf{x})$, the force vector $\mathbf{f}$ and the displacement vector $\mathbf{u}$. Since we want to optimize multiple different load cases, each representing one specific requirement, we consider ${\mathbf{f}}_{l};l=1,\dots , {n}_{\mathrm{l}}$ different load vectors, where ${n}_{\mathrm{l}}$ is the number of load cases. Due to different trim-body mass conditions for each discipline it may also be necessary to use different FE-models, each represented by one specific stiffness matrix ${\mathbf{K}}_{m}\left(\mathbf{x}\right);m=1,\dots , {n}_{\mathrm{m}}$ where ${n}_{\mathrm{m}}$ is the number of FE-models. Then, the overall optimization problem can be formulated as Multi Model Optimization (MMO) problem as follows:

2 $$\begin{gathered} \quad \quad \min f\left( {{\mathbf{u}}_{m} , {\mathbf{x}}} \right);\quad x \in {\mathbb{R}}^{{n_{{\text{d}}} }} \hfill \\ {\text{s.t.}}\quad g_{j} \left( {{\mathbf{u}}_{{\mathbf{m}}} , {\mathbf{x}}} \right) \le 0;\quad j = 1, \ldots , n_{{\text{c}}} \quad \quad \hfill \\ \quad \quad x_{i}^{{\text{L}}} \le {\mathbf{x}}_{i} \le x_{i}^{{\text{U}}} ;\quad i = 1, \ldots , n_{{\text{d}}} \hfill \\ \end{gathered}$$

where ${\mathbf{u}}_{m}$ results of

$${\mathbf{K}}_{m} \left( {\mathbf{x}} \right){\mathbf{u}}_{m} = {\text{w}}_{l\left( m \right)} {\mathbf{f}}_{l\left( m \right)} \quad m = 1, \ldots , n_{{\text{m}}}$$

and $l(m)$ maps the $l$-th load case to its corresponding FE-model $m$. Here, $f({\mathbf{u}}_{m},\mathbf{x})$ defines the objective function, ${g}_{j}({\mathbf{u}}_{m},\mathbf{x})$ are the inequality constrains, ${x}_{i}^{\mathrm{L}}$ and ${x}_{i}^{\mathrm{U}}$ are the $i$th design variable’s lower and upper bounds, respectively. The weighting factor ${\mathrm{w}}_{l(m)}$ is introduced for the purpose of balancing the different load cases and to derive a variety of generative design proposals. As can be seen, in this MMO framework the design variables are the coupling parameter between each load case, model or discipline. The load cases can be grouped as explained in the following:

### Load cases from static, driving dynamics, NVH, and crash

#### Statics, driving dynamics and NVH

**Figure 5:**
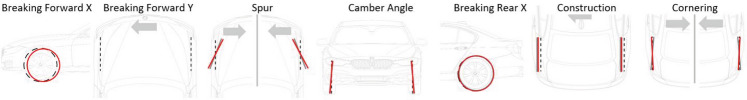
Example of driving loading conditions and respective measurements of stiffness.

To measure global stiffness in bending and torsion multiple load cases are defined, each focusing on a specific behavior of the vehicle in certain driving conditions. Some load cases are defined which use inertia relief approach, representing driving conditions like cornering, breaking among others as illustrated in Fig. 5.

**Figure 6:**
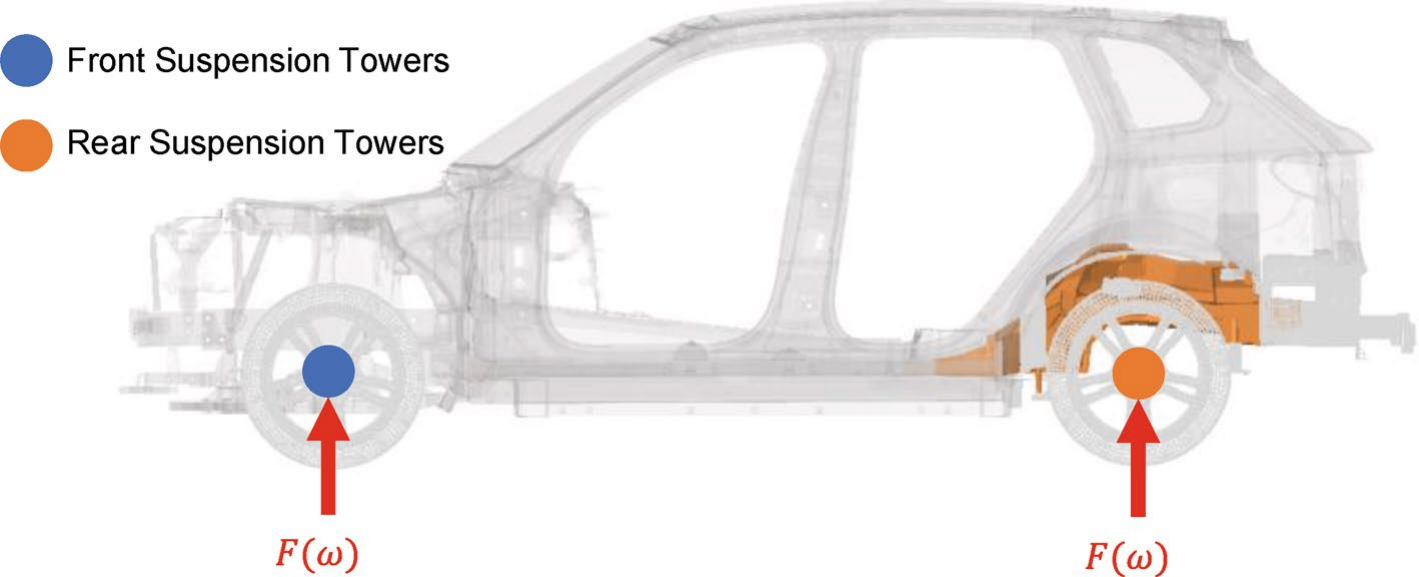
NVH load case: excitation of front and rear suspension towers.

An exemplary NVH load case is illustrated in Fig. 6, which is used to assess road NVH performance. Here, the four front and rear damping towers are actuated by dynamic forces $F\left(\omega \right)=Fsin(\omega t)$.

**Figure 7:**
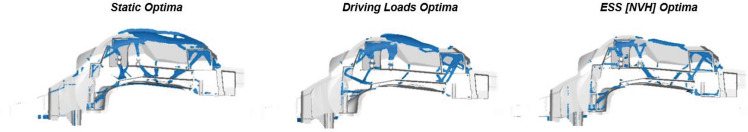
Topology optimization results for static, driving dynamics loads and NVH.

It is important to note that static, driving conditions and NVH load cases provide important input for the design of the casting component, however including only these loads would lead to topology results which would not satisfy all requirements. Figure 7 illustrates topology results for individual load cases. The results show load-paths that satisfy only a reduced number of targets.

#### Crash

**Figure 8:**
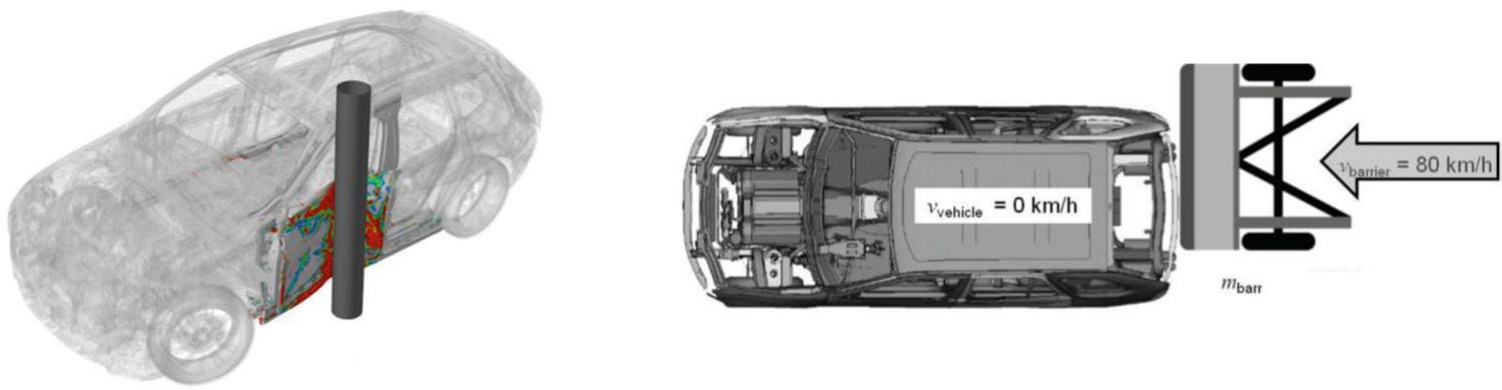
Driving crash load cases: pole-side-impact (left) and rear-impact (right).

To ensure passive vehicle safety, OEMs must fulfill a series of requirements within predefined test situations from a legal perspective. In addition, to achieve the overall goal of passive safety, OEMs typically define and verify their own validated measures. In the following some driving crash load cases are shortly explained and illustrated schematically in Fig. 8.

On the left a pole-side-impact is shown. Here, the car collides sidewise with a rigid pole with an initial velocity of 32 km/h. This simulates the collision with roadside objects such as trees. The pole’s intrusion into the car should be limited to protect the occupants and to prevent damage to other automotive parts, such as battery and fuel tank.

A second driving crash load case is the high-speed rear-impact (Fig. 8, right), where the car is hit from behind by another car or a rigid barrier with an initial velocity of 80 km/h.

Such crash tests come with large deformations, irreversible structural changes and contact within the structure itself and with the impactors. Mechanically, this reflects a transient problem which involves dynamic effects, nonlinearities in geometry, material, and due to contact. As a result, the computational costs for analyzing the structural responses increase massively. Explicit time integration schemes are usually employed. In that case sensitivities cannot be calculated as efficiently as it is the case for problems of linear nature. Consequently, for the optimization of such problems, gradient free optimization methods like evolutionary algorithms^22–24^ or Response Surface Methods (RSMs) like for example neural networks^25,26^, kriging^27–30^, or Radial Basis Functions (RBF)^31^ are often used. For a comparative review on RSMs please refer to Fang et al.^32,33^ or to Büttner et. al.^11^, addressing the challenges related to car body development. Unfortunately, these methods all combine a strong limitation regarding the number of design variables ${n}_{\mathrm{d}}$, since the computational effort significantly increases with ${n}_{\mathrm{d}}$. Hence, topology and free-sizing applications involving a huge number of design variables are not applicable using such methods.

For those reasons nonlinear dynamic response topology optimization still represents a challenging field of research. State of the art methods to name are: the Graph and Heuristic-based Topology optimization (GHT) approach, where cross-section of profiles^34^ are optimized using two dimensional graphs. This approach is limited to the application of extruded profile structures. However, currently efforts are made to extend the methodology to three-dimensional structures^35^.

Moreover, Patel introduced the so-called Hybrid Cellular Automaton (HCA)^36,37^. This is a heuristic motivated approach which aims at homogenizing the strain energy density within the investigated structure and mimics the growth of bones. For this purpose, the Cellular Automaton (CA) paradigm^38^ is combined with FEM and the SIMP approach.

The definition of linear auxiliary load cases enabling linear static response optimization is another prominent technique for overcoming the missing sensitivity issue. The nonlinear dynamic optimization problem then is split into an analysis domain where nonlinear dynamic analysis is performed and a design domain where a set of linear static response optimization subproblems is solved afterwards. The Equivalent Static Load (ESL) method is a well-known technique for calculating such auxiliary load cases. Numerous examples of effective applications in sizing, shape, free-sizing, and topology optimization exist^39–47^, but the method also suffers from limitations, e.g. referring to Refs.^48,49^. The ESL-procedure has recently been extended by the introduction of the so-called DiESL-method for sizing and topology optimization problems, incorporating nonlinearities in geometry and material. It reportedly results in better approximations in the linear static response sub-problem for nonlinear dynamic problems such as crash^48,50^.

Another familiar approach is to use physically motivated ESLs^51^. The ESLs are then not calculated in a mathematically rigorous way, but by following physical considerations for instance the necessary force level to attribute the kinetic energy needed to be absorbed during crash. Even more straight forward is to directly transfer contact forces measured during crash analysis to the linear auxiliary load cases, as recently demonstrated by Ref.^52^.

To enable incorporating the nonlinear dynamic crash load cases into the optimization framework specified in Eq. (2), in a similar fashion as in the ESL approaches, the crash load cases are linearized as will be explained in the chapter 0.

### Linearization techniques

To integrate the Crash and NVH load cases into the MMO framework (Eq. 2) a pragmatic approach is needed.

For the rear-impact load case (Fig. 8, right), the linearization is performed as explained in the following. Linear loads are applied to the structure such that the resulting deformation modes are similar to those of the nonlinear dynamic crash case. In detail, the high speed crash event is split into different stages. For each of these stages, nodal loads measured at defined sections during the crash event are transferred to define linear static auxiliary load cases. Afterwards the strain energy distribution and global deformation shape is compared visually between the actual crash simulation and the linear static auxiliary load cases and trigger loads are added to increase similarity.

**Figure 9:**
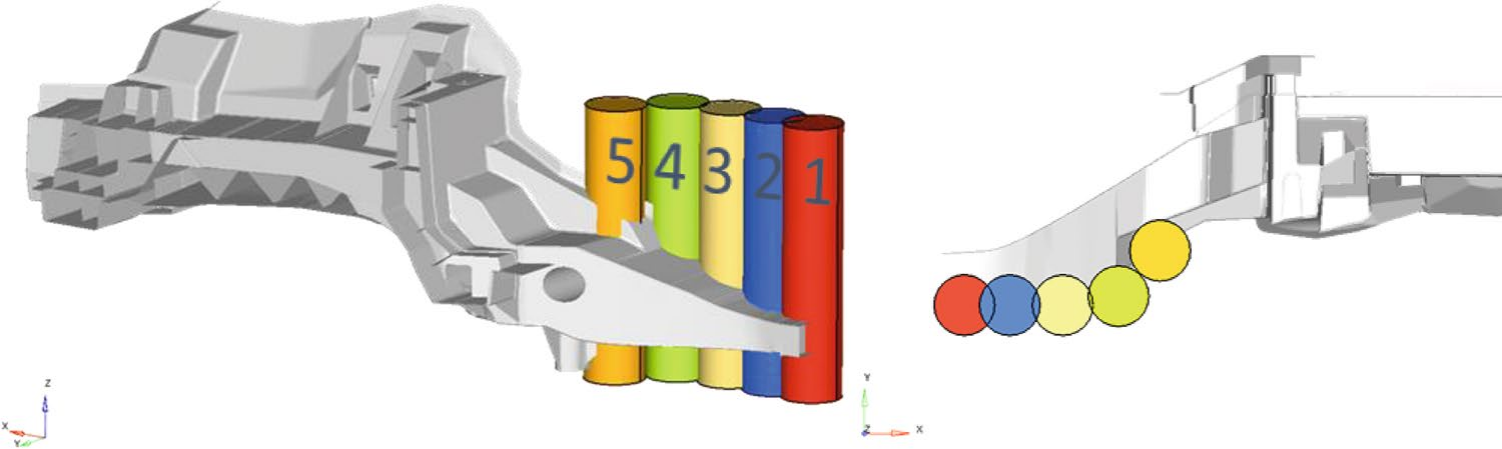
Different pole positions for pole-side-impact.

The pole-side impact load case is linearized in a similar way. Here, the contact force between pole and structure is measured during the nonlinear dynamic analysis and transferred to create linear auxiliary load cases. This is done for different impact positions of the pole as illustrated in Fig. 9. The loads derived for each pole position are then balanced, such that the resulting displacements at each pole position decrease monotonically from pole position 1–5. This aims at deriving a homogenous stiffness distribution over the components front horns, to prevent crack-initiation due to high differences in stiffness along this end.

To derive a linearized equivalent NVH load case, the frequency domain is linearized to only 2 Hz free structure to eliminate boundary effects at the supports. Basic static stiffness is derived at the attachment points where in reality a dynamic load stemming from road-wheel dynamics or engine excitation arises.

It’s important to note that the original NVH and crash load cases are driven by nonlinear effects. Even if the linearized loads yield similar deformation modes or responses for a specific design, one could not expect equally good consistency if the design is changed. The topology optimization targets to achieve good load-paths. The resulting load-paths are then optimized using RSMs, where high frequency or such design dependent nonlinear effects can be incorporated based on a smaller number of design variables.

### Multidisciplinary combined topology and free-size optimization

Finally for every discipline a linear auxiliary load case is available enabling combined free-size and topology optimization. As a next step, constraints accounting for castability are introduced and the linearized load cases are balanced among each other.

#### Casting constraints

**Figure 10:**
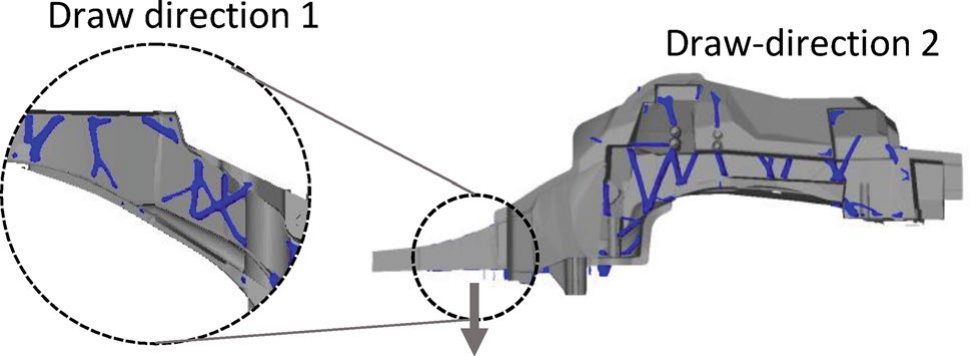
Draw direction constraint to account for castability of ribs.

To integrate manufacturability into the optimization process, some casting constraints are defined. First, draw directions are defined, such that the mold can be removed after solidification. Figure 10 illustrates two different draw directions set up.

**Figure 11:**
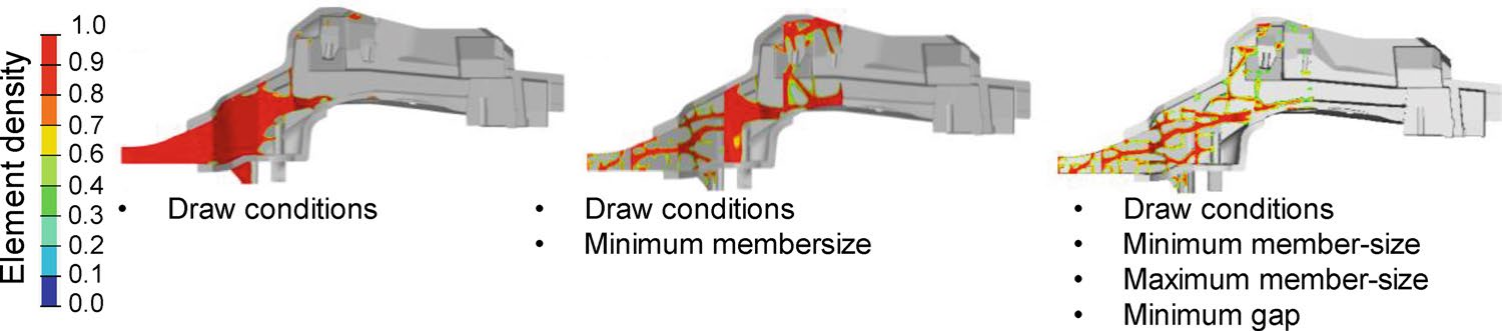
Resulting topology depending on employed manufacturing constraints.

Second, minimum member-size and maximum member-size constraints are introduced, defining the minimum and maximum diameter of members to be formed during topology optimization, respectively. Third, a minimum gap constraint is defined, which defines the minimum spacing between structural members formed, in order to avoid tool overheat. The impact of such constraints on the resulting topology is exemplified in Fig. 11.

#### Balancing of load cases

**Figure 12:**
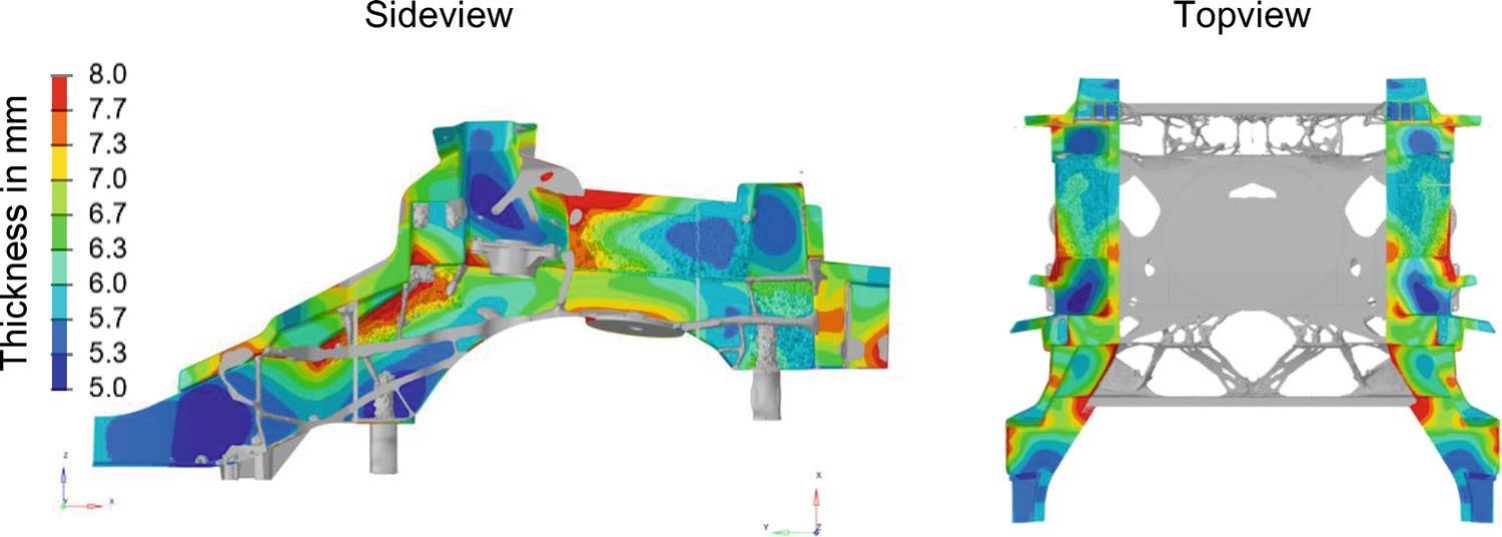
Individual result of combined free-size and topology optimization for rear-impact load case.

Now the different loads ${\mathbf{f}}_{l}$ are balanced to drive adequate results, i.e. the weighting factors ${\mathrm{w}}_{l}$ are determined, especially for the simplified linearized loads. For this purpose, the mass is minimized, and the results are considered adequate if the resulting mass is close to a predefined reference value. Optimizing each load case individually first can be very helpful, as the resulting topology is representative for the load cases or disciplines (e.g. NVH, crash, driving dynamics) sensitivities. If the resulting mass of such an individual optimization is considerably smaller than the reference value, the corresponding loads can be increased. As an example, the combined free-size and topology optimization result of the rear-impact load case is given in Fig. 12. Here, the resulting mass exceeded the reference value, which is why the corresponding weighting factor has been decreased. This procedure results in many different design proposals, each of which can be evaluated based on the requirements related to all load cases.

### Selection of best candidate and realization

**Figure 13:**
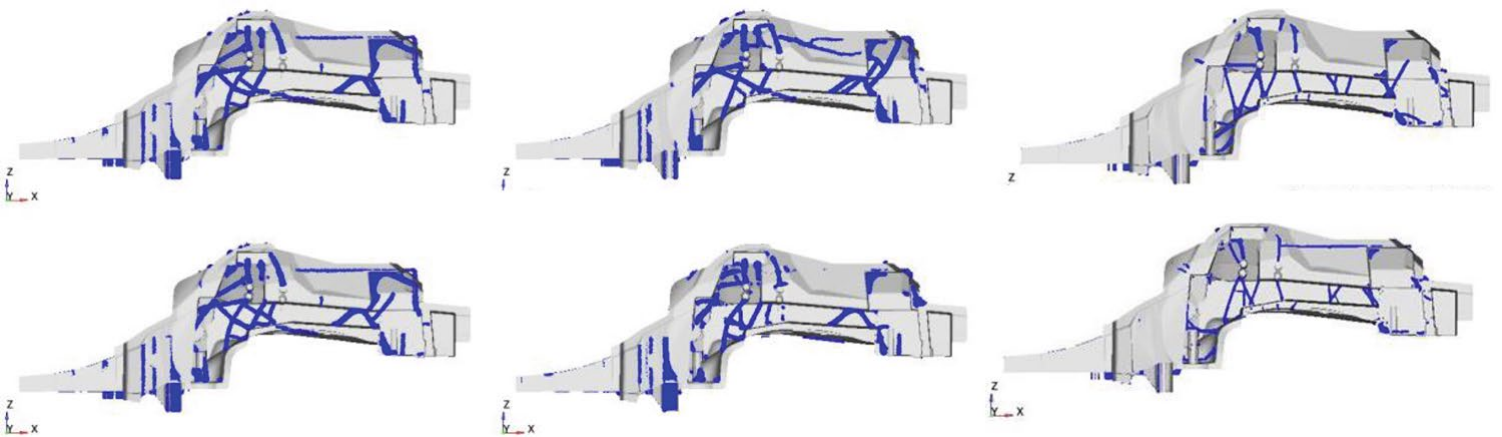
Different rib designs resulting of load balancing process using linearized models.

**Figure 14:**
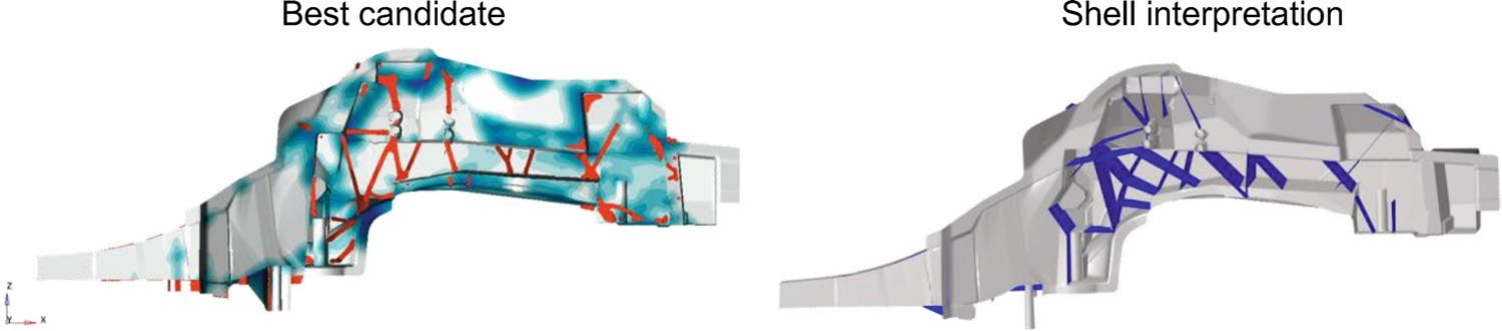
Best candidate design and corresponding shell realization.

Figure 13 shows some of those design proposals. Based on the requirements fulfillment and the overall resulting mass a best candidate design is selected (Fig. 14, left). The rib structure is then transferred to a shell structure (Fig. 14, right). This shell design can be parameterized by a small number of design variables specifying the ribs height and thickness.

The resulting shell interpretation is then again evaluated based on casting, crash and NVH simulation. Eventually, ribs are added manually to improve the casting component’s performance. For example, some ribs have been added to enhance the flow of the liquid metal into the component’s front horns. After each of those adaptions a free-size optimization is run based on the linearized models where the overall mass is minimized again. This aims at rebalancing the structural members based on the new design in the interest of mass minimization and optimal structural performance.

Finally, the shell realization illustrated in Fig. 14, right is derived. This realization is parameterized by a small number of design variables only, such that RSM-based optimization can be used to further optimize the structure. Please note: the actual purpose of creating the linearized models is fulfilled with the realization of the shell model. The model is nevertheless further exploited: each adaption resulting of the RSM-based optimization can be fed back into the linearized models containing all requirements for the purpose of balancing thicknesses between all loads and disciplines as explained above.

## Response-surface-method-based crash optimization using machine learning to emulate engineering expertise

RSM or also known as surrogate-based optimization approaches are the foundation of phase-two of the generative design approach illustrated in Fig. 3. They are frequently used to solve crash optimization problems as they provide a controlled manner of managing the required computational budget by creating mathematical models that are used in lieu of the finite element solver. Such approaches require the translation of engineering requirements into scalar quantities that can be constrained by the optimizer. However, often it is not enough to use only scalar quantities to represent the complex requirements imposed on the crash results.

Typical crash constraints include time dependent measurements of forces, displacements, strains, damage, distributed energy absorption, etc. The time needed to define such constraints is extensive and the number of iterations needed between optimization experts and crash design experts limits its application in the fast-paced development process of BIW structures.

As a result, multiple processes rely on manual validation trough visual inspection of individual post-processed simulation results. An expert with a trained eye can quickly determine whether the crash behavior meets the requirements or not. This is particularly true for more sophisticated result fields visualized by contour plotting directly on the considered geometry, like stress or plastic strain fields. Damage and failure of material play an important role in crash design to absorb kinetic energy during the crash event. In state-of-the-art crash modelling sophisticated failure models based on nonlinear material laws are applied. For aluminum die casting the brittle material behavior makes it inevitable to differentiate failure between local cracking and global structural collapse. Such a distinction is very difficult to be captured by only scalar value results, it rather represents a classification type of interpretations. Thus, the classical response-surface-based approach based on a regression of continuous scalar results like intrusion, load level, or max plastic strain, needs to be enhanced by means of classifying a result into local acceptable failure or global collapse. This calls for incorporation of machine learning into the optimization process as described in the following. We therefore mimic the decision-making process of an expert through the use of clustering and classification on top of the widespread regression in an expert emulation process^53^.

### RSM-based optimization

Consider a general optimization problem in the form:

3 $$\begin{gathered} \quad \quad \min f({\mathbf{x}});\quad \quad x \in {\mathbb{R}}^{{n_{{\text{d}}} }} \hfill \\ {\text{s.t.}}\quad g_{j} \left( {\text{x}} \right) \ge 0;\quad \quad j = 1, \ldots ,{ }n_{{\text{c}}} \hfill \\ \quad \quad x_{i}^{{\text{L}}} \le {\text{x}}_{i} \le x_{i}^{{\text{U}}} ;\quad i = 1, \ldots ,{ }n_{{\text{d}}} \hfill \\ \end{gathered}$$

**Figure 15:**
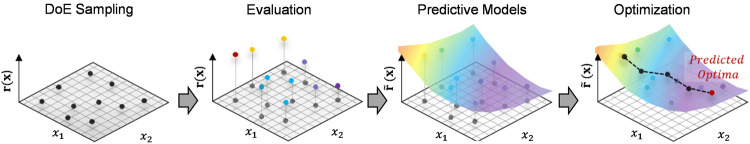
RSM-based optimization process.

Such problems can be solved using direct optimization methods such as the Method of Feasible Directions (MFD)^54^ or Sequential Quadratic Programming (SQP)^55^. However, for problems that are very noisy and/or for which analytical gradients are not available, best practice dictates the use of surrogate based optimization. Although there are many methods proposed in literature, and many review articles written on the subject^56–60^ the process usually consists of a combination of the same four key ingredients shown in Fig. 15.

#### Design of experiments (DOE)

Test plan of which points should be investigated within the space of the design variables. There are many types of DOE methods available, but it is generally advisable to use one that uniformly distributes points within the space spanned by the design variables.

#### Evaluation

Evaluation of the responses at each of the DOE points. This is where most of the computational effort is spent. All DOE points are independent which means they can be run in parallel, often using High Performance Computing (HPC) facilities with many cores.

#### Predictive modelling

Training of predictive models of the responses as function of the design variables. Generally, the training is done using a subset (here labeled training set) of the DOE points so that the remaining points (here labeled the test set) can be used to evaluate the quality of the predictive model.

#### Optimization

Finally, an optimization algorithm is used to find the predicted optimum using the predictive models in lieu of evaluating the true responses. The optimization problem (Eq. 3) then becomes:

4 $$\begin{gathered} \quad \quad \min \tilde{f}({\mathbf{x}});\quad \quad x \in {\mathbb{R}}^{{n_{{\text{d}}} }} \hfill \\ {\text{s.t.}}\quad \tilde{g}_{j} \left( {\mathbf{x}} \right) \ge 0;\quad \quad j = 1, \ldots , n_{{\text{c}}} \hfill \\ \quad \quad x_{i}^{{\text{L}}} \le {\text{x}}_{i} \le x_{i}^{{\text{U}}} ;\quad i = 1, \ldots , n_{{\text{d}}} \hfill \\ \end{gathered}$$

where ˜ denotes a predicted quantity.

Due to the speed at which the predictive model can be evaluated, the number of points needed to reach a conclusion is secondary to how efficiently the method can find multiple optima in complex spaces. It is common to verify the predicted optima through evaluation of the FE-model after which the process can be repeated several times until satisfactory results are obtained.

#### Clustering

**Figure 16:**
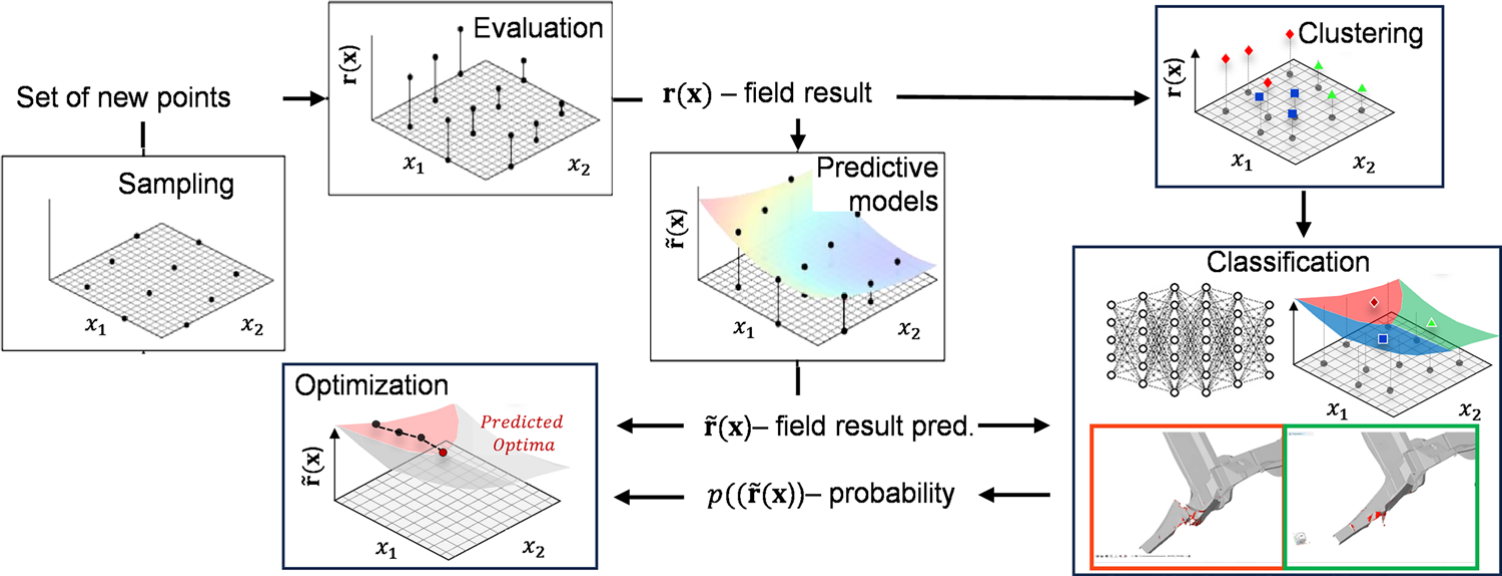
RSM-based optimization process including expert emulation.

The expert emulation process is incorporated into the RSM-based optimization process as illustrated in Fig. 16. As in the traditional surrogate based optimization process, the process begins by DOE sampling and evaluation of each point in the test plan. After this, each DOE point needs to be labeled as per the visual inspection of the expert. Rather than requiring the expert to manually do a visual inspection and labeling of each run, clustering is used to automatically group runs with similar results, denoted $\mathbf{r}(\mathbf{x})$, together. This results in ${n}_{\mathrm{g}}$ different groups or clusters of results.

#### Classification

Once all runs have been labelled, a classifier can be generated. The classifier is meant to mimic a visual inspection of the postprocessed simulation result fields by an expert user. The classifier is therefore trained using a result field $\mathbf{r}(\mathbf{x})$ as the input and the label of each run as output. The classifier can be denoted as

5 $$\mathbf{p}\left(\mathbf{r}\left(\mathbf{x}\right)\right)$$

where $\mathbf{p}{\left(\mathbf{r}\left(\mathbf{x}\right)\right)}^{T}=\left({p}_{1}\left(\mathbf{r}\left(\mathbf{x}\right)\right),\dots , {p}_{{n}_{\mathrm{g}}}\left(\mathbf{r}\left(\mathbf{x}\right)\right)\right)$ is a vector and ${p}_{{n}_{\mathrm{g}}}\left(\mathbf{r}\left(\mathbf{x}\right)\right)$ gives the probability that $\mathbf{r}\left(\mathbf{x}\right)$ belongs to the group ${n}_{\mathrm{g}}$.

#### Optimization based on expert emulation

To determine the optimum design belonging to a certain group corresponding to a favored structural behavior, the classifier can be used within a surrogate based optimization framework:

6 $$\begin{gathered} \quad \quad \min \tilde{f}({\mathbf{x}});\quad \quad \quad \,x \in {\mathbb{R}}^{{n_{{\text{d}}} }} \hfill \\ {\text{s.t.}}\quad \tilde{g}_{j} \left( {\mathbf{x}} \right) \ge 0;\quad \quad \quad \,j = 1, \ldots ,{ }n_{{\text{c}}} \hfill \\ \quad \quad p_{l} \left( {{\tilde{\mathbf{r}}}\left( {\mathbf{x}} \right)} \right) \ge p_{l}^{{{\text{min}}}} ;\quad l = 1, \ldots ,n_{{\text{g}}} \hfill \\ \quad \quad x_{i}^{{\text{L}}} \le {\text{x}}_{i} \le x_{i}^{{\text{U}}} ;\quad \quad i = 1, \ldots ,{ }n_{{\text{d}}} \hfill \\ \end{gathered}$$

where the resultant field is determined using the RSM itself and ${p}_{l}^{\mathrm{min}}$ denotes the minimum required probability that the result belongs to the $l$-th group.

## RSM Optimization of pole side-impact enhanced by expert emulation for casting parts

**Figure 17:**
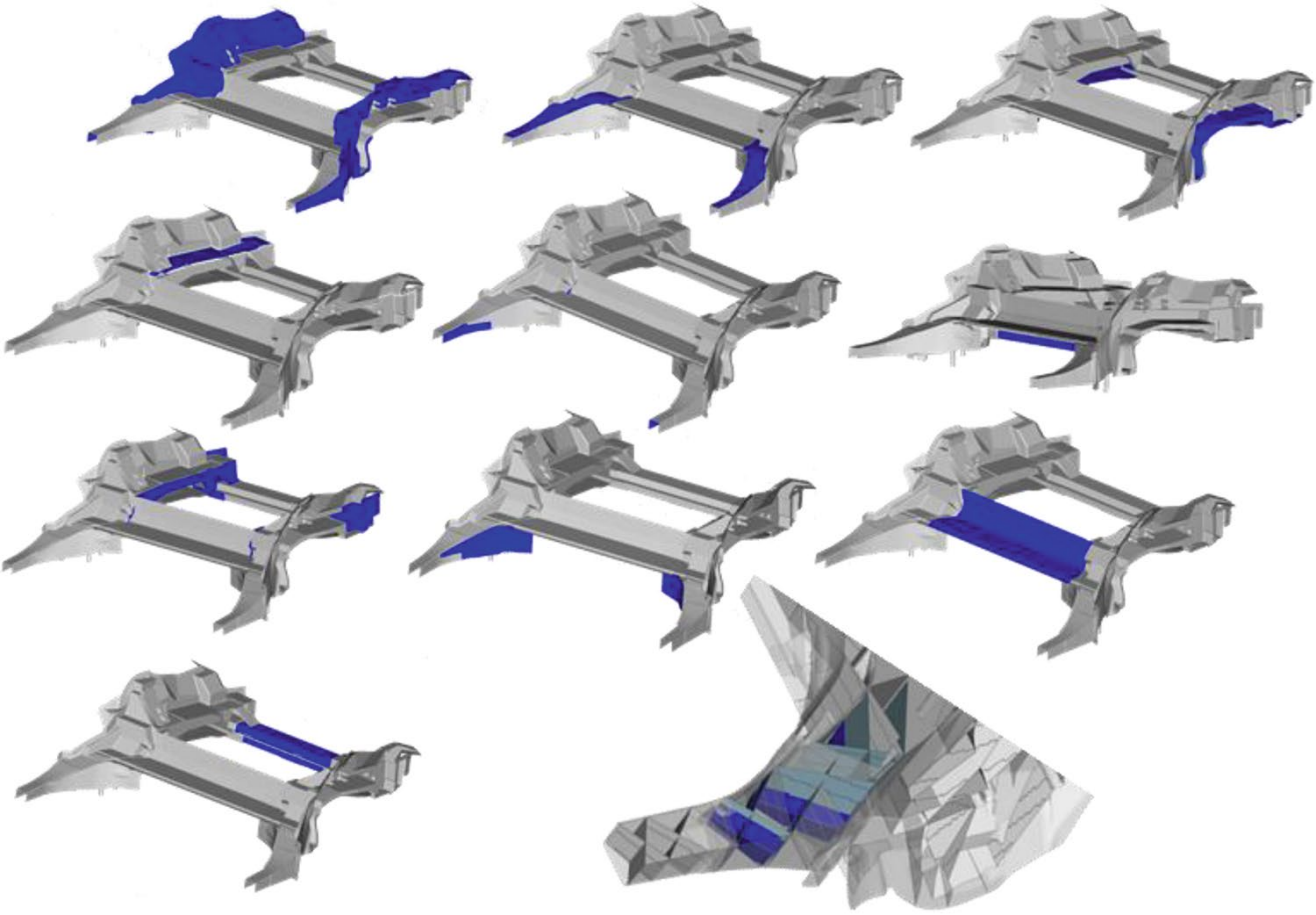
Shell sizing parameterization using about 20 design variables (blue).

As part of the second phase of the generative design process (Fig. 3) we apply RSM-based optimization to fulfill pole side-impact performance with minimal weight. As a first step the overall mega-casting part is optimized. The design variables have been chosen as representative thicknesses of key structural sections of the casting part including individual ribs, as outlined in Fig. 17. This defines a typical design space of about 20 design variables. As a second step the ribs inside of the front-horn region are fine-tuned to attribute their superior contribution to pole-side impact performance.

**Figure 18:**
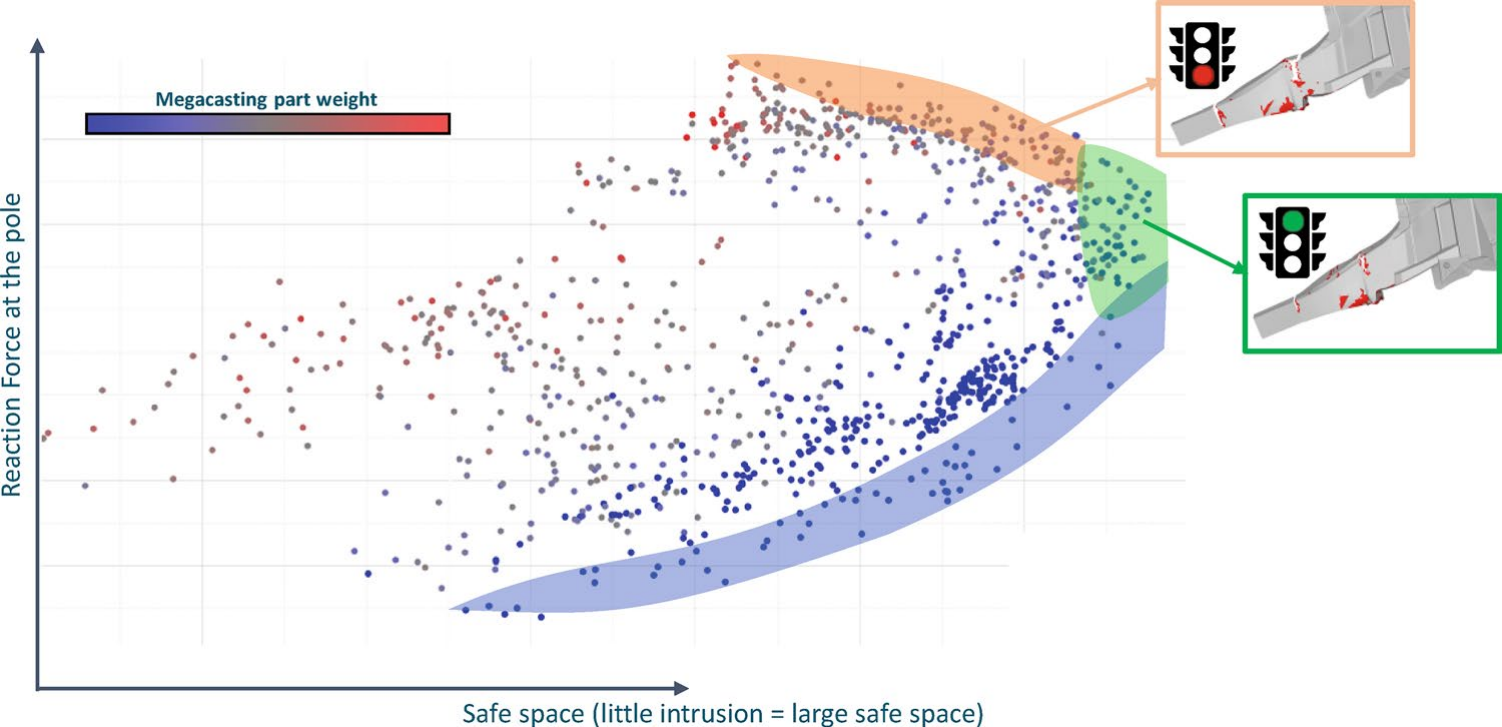
Pareto-optimal designs for the objectives maximum reaction force over safe space. Exemplary illustration of the two clusters integer (green) and collapse (orange).

The maximum reaction force at the intruding pole over time and the minimum remaining safe space are used as key performance indicators. In that safe space for instance passengers or battery modules are safe from impact by the pole. Figure 18 illustrates the pareto-optimal region maximizing safe space and maximum reaction force for one distinct pole position. Such region can be determined using e.g. the Global Response Search Method with Altair HyperStudy^61^. We see that there is a limit region to achieve all targets, highlighted in green. Where the blue region represents the limit to achieve safe space with minimal structural weight, the orange region represents the limit of the structure’s resistance to the pole impact loading. However, this orange color region compromises the safe space because due to broad material failure, or even partial structural collapse of the aluminum casting, the pole intrudes further. This region is dominated by designs representing a full collapse such as illustrated in the orange frame of Fig. 18. The green region is represented mostly by designs where only local failure occurs and the structure remains integer, as exemplarily shown in the green frame of Fig. 18. Such designs are key to balance energy absorption and intrusion and represent therefore the design target. It is important to note that even if those regions are dominated by one distinct structural behavior, the regions do mix up and it is very challenging to identify the best compromise design between minimum remaining safe space and maximum reaction force just based on this scalar results.

This is complicated by the necessity of achieving robustness. To derive a robust structure, one could vary the pole’s impact location along the side of the vehicle, as depicted in Fig. 9, and perform an optimization based on multiple load cases. Then, a scalar KPI based on all load cases has to be found, e.g. average minimal safe space over all pole positions, to target one robust optimum. Material failure could also be considered within the optimization problem, constraining e.g. the number of failed elements. No matter how, the quality of resulting optimal designs heavily depend on whether the chosen scalar measurements capture the engineering design target adequately or not.

For both above reasons, each design, has to be assessed by an expert individually. Depending on the number of simulations to be evaluated this process can vary from very time consuming to impractical. To circumvent this, clustering and derived classification constraints as explained in Section "Optimization based on expert emulation" are used. As representative cluster data the displacement fields, or the specific material failure contour fields, at the last time-step of the crash analysis are used. This resembles the same contour crash domain experts would look at while evaluating one single design. The classifier is trained using an initial DoE consisting of about 150 simulations. Then, the mass can be minimized while constrained on designs representing the targeted green (structurally integer) failure modes for various pole positions.

**Figure 19:**
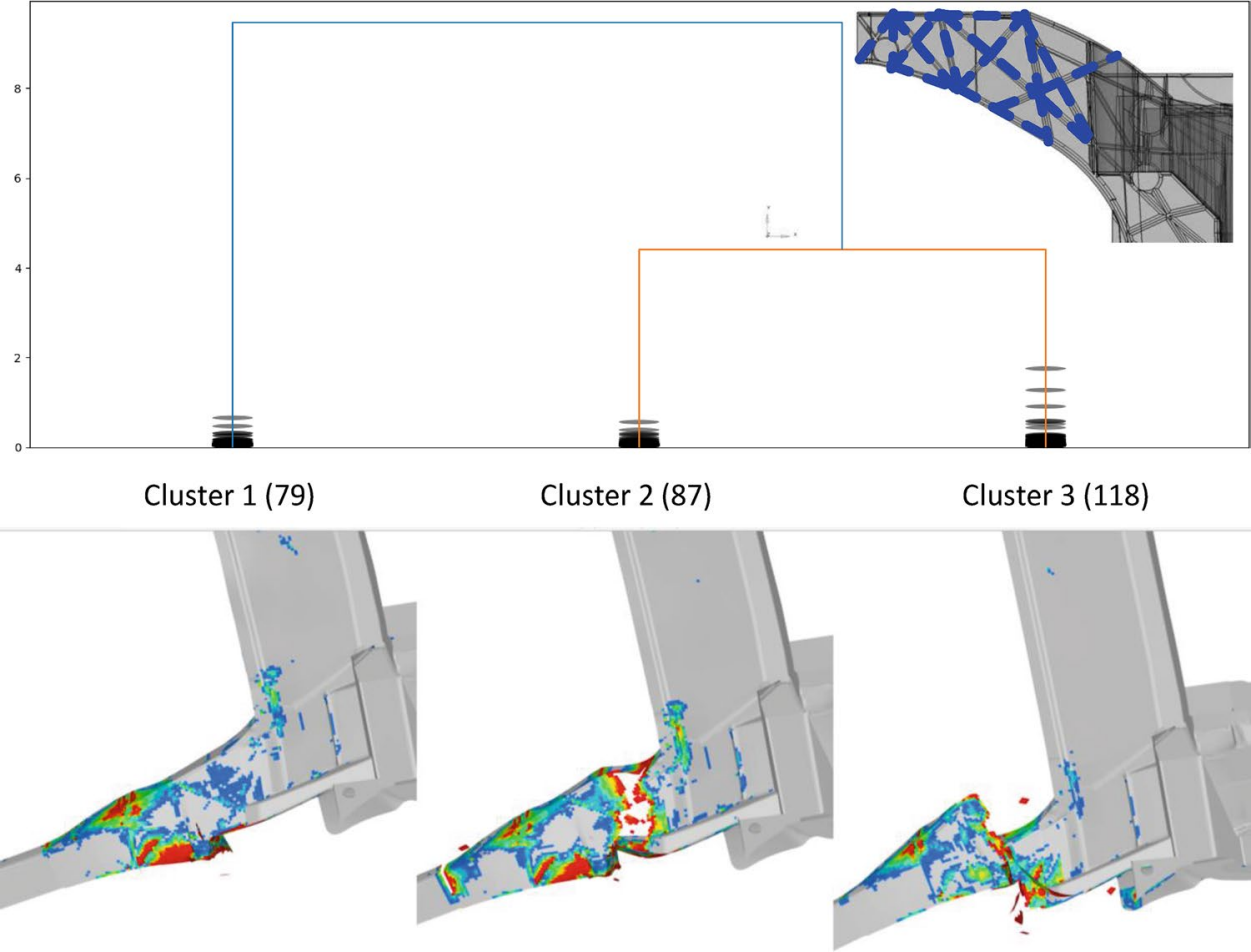
Clustering result of local horn-section RSM-optimization. Top-right insert sketches the rib thickness design-space in the local horn section. The dendrogram on top shows three clusters based on material failure contour fields of last time steps of 284 samples, respectively. One exemplary contour plot for each cluster is depicted below, visualizing the significantly different failure modes identified by unsupervised clustering: Cluster 1 = local failure only; Cluster 2 = structural shear collapse mode 1; Cluster 3 = structural shear collapse mode 2.

In a second RSM-based optimization phase we exploit this more detailed. Based on results of the first RSM optimization where the major thickness distributions of the structural sections are defined, this time only the local rib thicknesses of the horn region are considered. The results related to the most critical pole position are discussed as follows. Sampling and clustering of the crash results based on the failure contour at the last timestep reveals intriguingly different failure modes. We illustrate three different failure modes in Fig. 19. The dendrogram on top of the figure shows a separation of a first “cluster 1” with 79 results and two further separated clusters “cluster 2 [87 samples]” and “cluster 3 [118 samples]. Below the dendrogram we plot one individual sample of the respective cluster. Mind that within the respective clusters several sample results are collected. What can be observed is that the three clusters represent different failure modes where cluster 1 shows distributed local elemental failure and cracks, but no structural collapse of the horn section. In contrast, the other two clusters show more significant connected failure leading to shear-type separations of the horn section and the remaining casting structure. Obviously, the structural collapse shall be avoided for the optimal design. By analyzing the design variable setups corresponding to preferred cluster 1 results one identifies a particular design mechanism which consists of an inner section of the horn ribs where thicknesses are rather high and an outer section of ribs with a rather small thickness. This outer rib structural section serves energy absorption where the inner section stabilizes the overall structure. Minimizing the weight of the design space section and constraining the failure mode to preferred optimal cluster 1 results in a robust structural design of the local horn section for pole side crash. Feeding this design into the multidisciplinary overall design optimization enables favorable crash behavior, where traditional designs do not fulfill crash requirements due to brittle failure of aluminum structure.

## RSM-based optimization of casting process and part design

Next to the design challenge to fulfill structural requirements with minimal weight, the challenges related to the casting process are addressed using RSM-based optimization.

HPDC manufacturing processes are defined by three main injection phases, prefilling, filling and compaction. During prefilling, low velocity and a sophisticated runner system design enables appropriate inflow of the material at the ingates. When the material reaches the ingate, the second phase of the injection, the filling phase begins. The runner system is very narrow in the region in contact with the part, thus, material velocity increases highly in this region and enters the part at very high speed. This is an important requirement in HPDC processes to avoid premature solidification of the metal before the full component is filled. Lightweight designed parts contain very thin regions in which temperature decreases fast. To enable such lightweight design the filling time must always be lower than the most unfavorable solidification time.

Finally, during compaction phase, extra material is pushed into the cavity at high pressure to reduce shrinkage porosity and improve material characteristics such as strength and ductility, for which aluminum alloys nowadays are designed to fulfill high standards. This is especially driven by structural crash requirements. It is therefore key to design a casting process which robustly achieves required material quality particularly at the part region where crash requirements are essential. This represents a design problem coupling structural and process targets. In the following, focusing on the most critical filling phase, the problem is addressed by optimizing the ingate system and as well by considering part design bound to structural requirements within casting simulations.

### RSM-based optimization of inlet design

**Figure 20:**
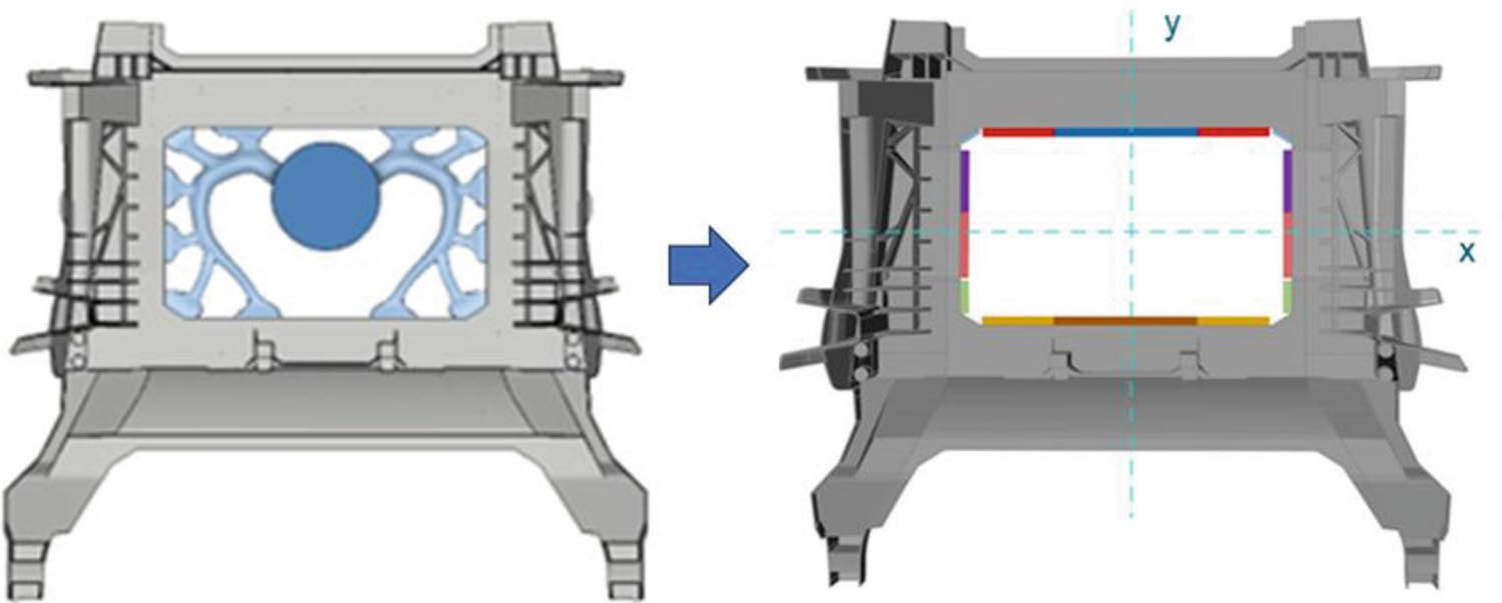
Ingate system simplification for parametrization.

To obtain a good quality casted part using HPDC it must be ensured that the component does not solidify before the die is filled and the last part to solidify must be the gate, to enable the entrance of material during the last phase. The design of the ingate system itself dictates initial flow conditions and therefore is key to achieve high quality casting. There is no clear mathematical relationship for the number of gates required for a die-casting part and rather empirical and rough assumptions are typically taken in industry^62^. In the following RSM-based optimization is employed to determine the best configuration that minimizes the number of ingates while achieving the shortest filling time. As illustrated in Fig. 20 the symmetric ingate system consists of 16 different ingates, parameterized by 8 discrete design variables (open or closed).

**Figure 21:**
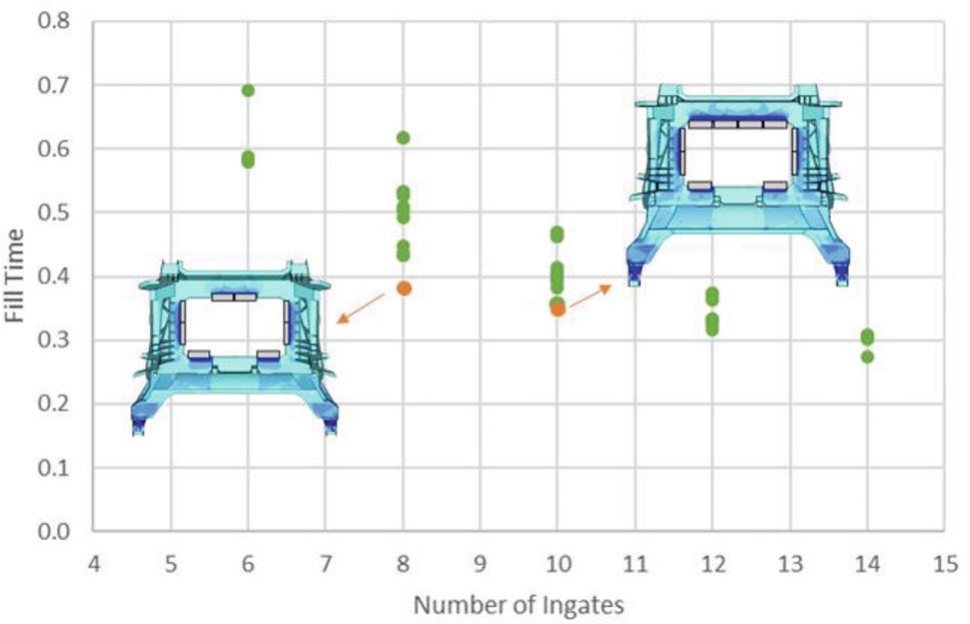
Filling times varying the number of ingates and their position.

The transient filling simulation is solved using an implicit Eulerian manufacturing solver that couple and solves the governing equations for fluid flow and heat transfer. A DOE was conducted with over 150 runs. The analysis focused on the impact of the number of inlets (ranging from 6 to 14). One key finding from the study, as depicted in Fig. 21, is that there is a specific position where the filling time for a configuration with a higher number of ingates is equivalent to one with two fewer ingates (Fig. 21, left). This demonstrates that while filling time is generally proportional to the number of ingates, the specific configuration and part geometry also play a significant role.

This observation indicates that achieving the same filling time can be accomplished with fewer ingates, resulting in less waste material in the ingate system. This finding highlights the importance of considering both the number and configuration of ingates to optimize the casting process effectively.

Besides, it is essential to consider the multidisciplinary performance requirements, specifically castability and crash performance. The front horns of the component are identified as the most critical region in both aspects. These horns play an important role in crash performance, and it is crucial to minimize defects in this area during the manufacturing process. For this purpose, a uniform filling process is targeted while minimizing the overall filling time and ensuring the last region to be filled is not the horns but the upper region.

**Figure 22:**
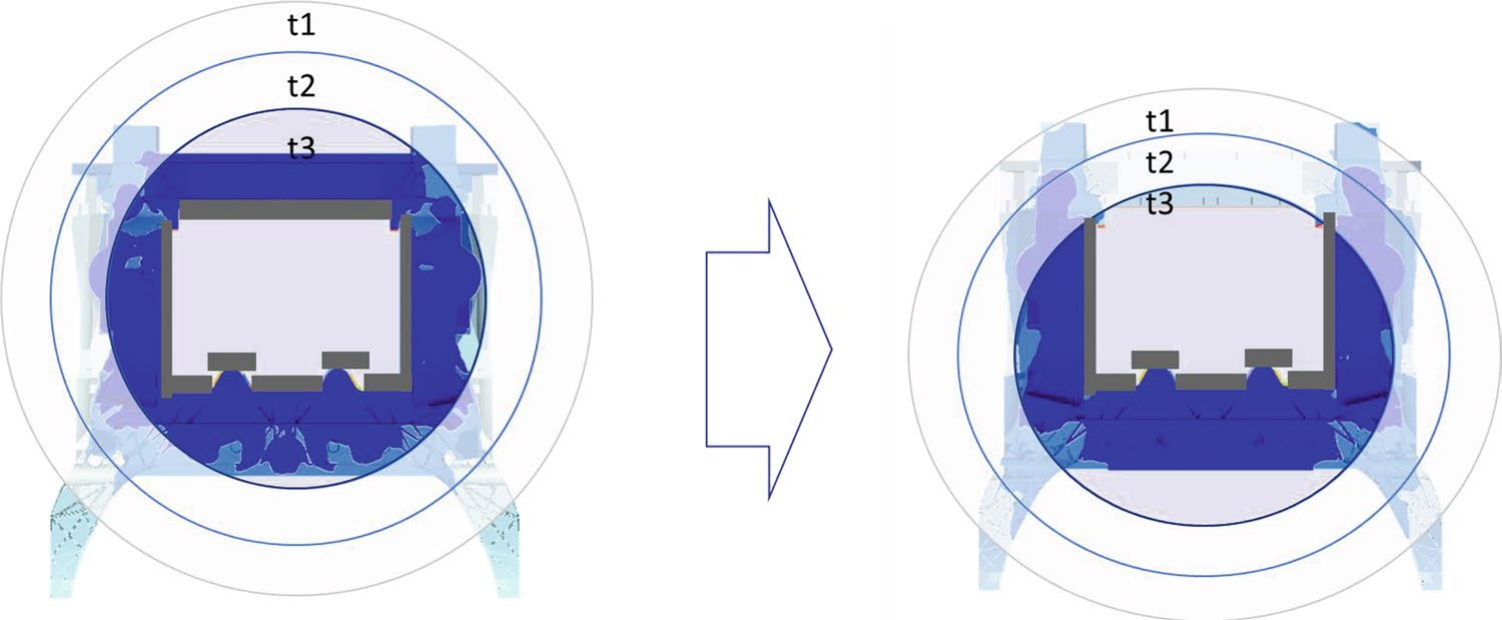
Ingate optimization initial and final state. Three material front timesteps t1, t2, t3 are represented by color. On the left the beginning of the filling (t3) is rather circular around the ingate section and the final material front (end of the filling, t1) is only located at the horns. In contrast on the right, the material front at the beginning (t3) is rather imbalanced favoring the lower section leading to a homogeneous material front at the end of the filling (t1). This shifts a potential risk zone of material defects from the structurally critical horn section to the upper crossbeam section.

Figure 22 shows the filling fronts at different times for the initial design with all ingates used on the left and for the optimal design on the right side. The optimal design differs to the previously found optimal design by using 2 ingates in the front instead of the rear. This accounts for early filling of the front horns but also balances the flow to be concentric to the ingates instead of displaced to the front.

### Multidisciplinary optimization approach to design mega-casting part w.r.t. structural and casting requirements

**Figure 23:**
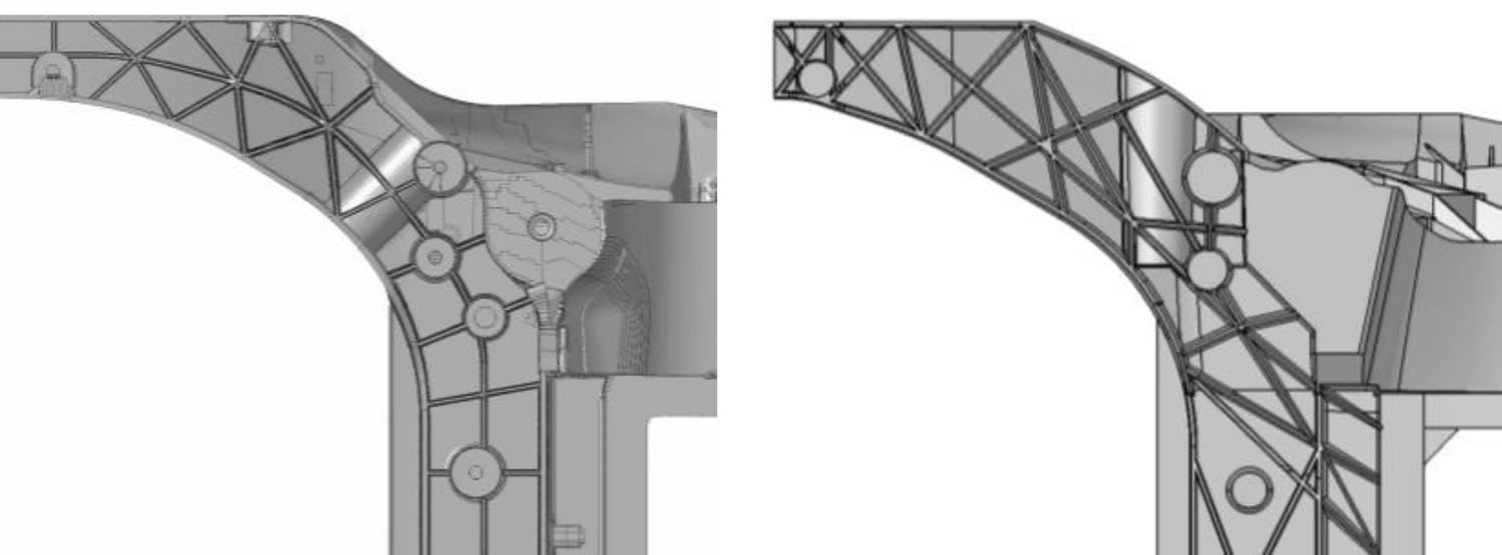
Comparison of rib topology and orientation in the critical pole side-crash section: on the left a classical design approach with regular pattern. On the right the proposed design optimizing filling together with crash performance.

We have demonstrated above how the overall ingate design can be adapted to optimize the filling process regarding structural and casting requirements. Additionally, local geometric part design also determines the filling in the region of the front horns. Ribs oriented from the ingate directly to the front section ease filling compared to for instance a regular square rib pattern, see Fig. 23. Also thicknesses of ribs and base surface affect the filling time and filling quality. As explained in chapter 0 this selection is critical to fulfill pole crash requirements and the thickness distribution has been optimized by RSM and clustering constraints. Only a specific thickness distribution fulfills crash requirements, where a soft section towards the pole allows energy absorption and a strong section of inner ribs stabilizes against structural collapse.

Based on the RSM approach to achieve optimal rib position, thickness, and base surface distribution to fulfill crash requirements, one can easily include the casting simulation of the part coupled to these same design variables. Coherence of the separate crash and casting simulations with their individual models and respective design parametrization is achieved by running both in parallel based on design variables coupling the equivalent geometric design freedom in both the crash and the casting model. Thus, a pareto front of targets stemming from crash and casting can be derived and weight optimal designs fulfilling all targets could be explored. We refer to^63^ where this proposed process has been evaluated for a simplified structure representing the coupled structural and casting process optimization.

## Validation of weight-optimal multi-disciplinary mega-casting design

**Figure 24:**
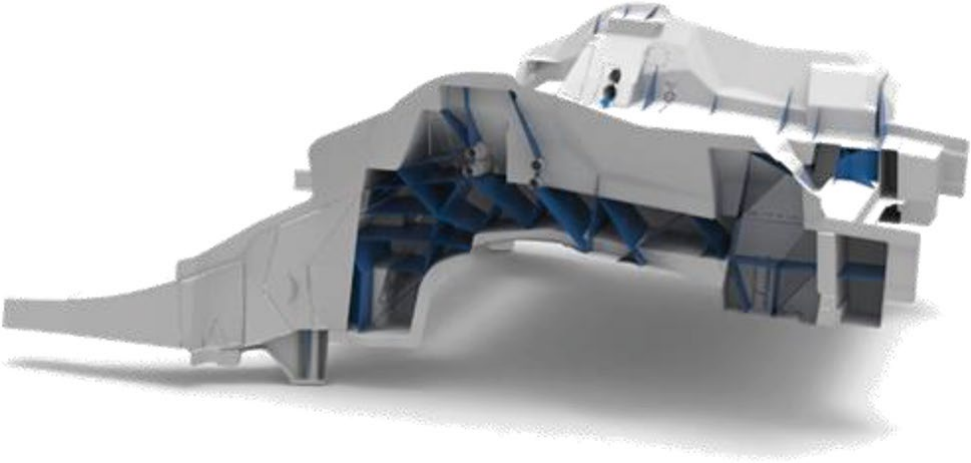
Final design resulting of two-phase generative design approach.

**Figure 25:**
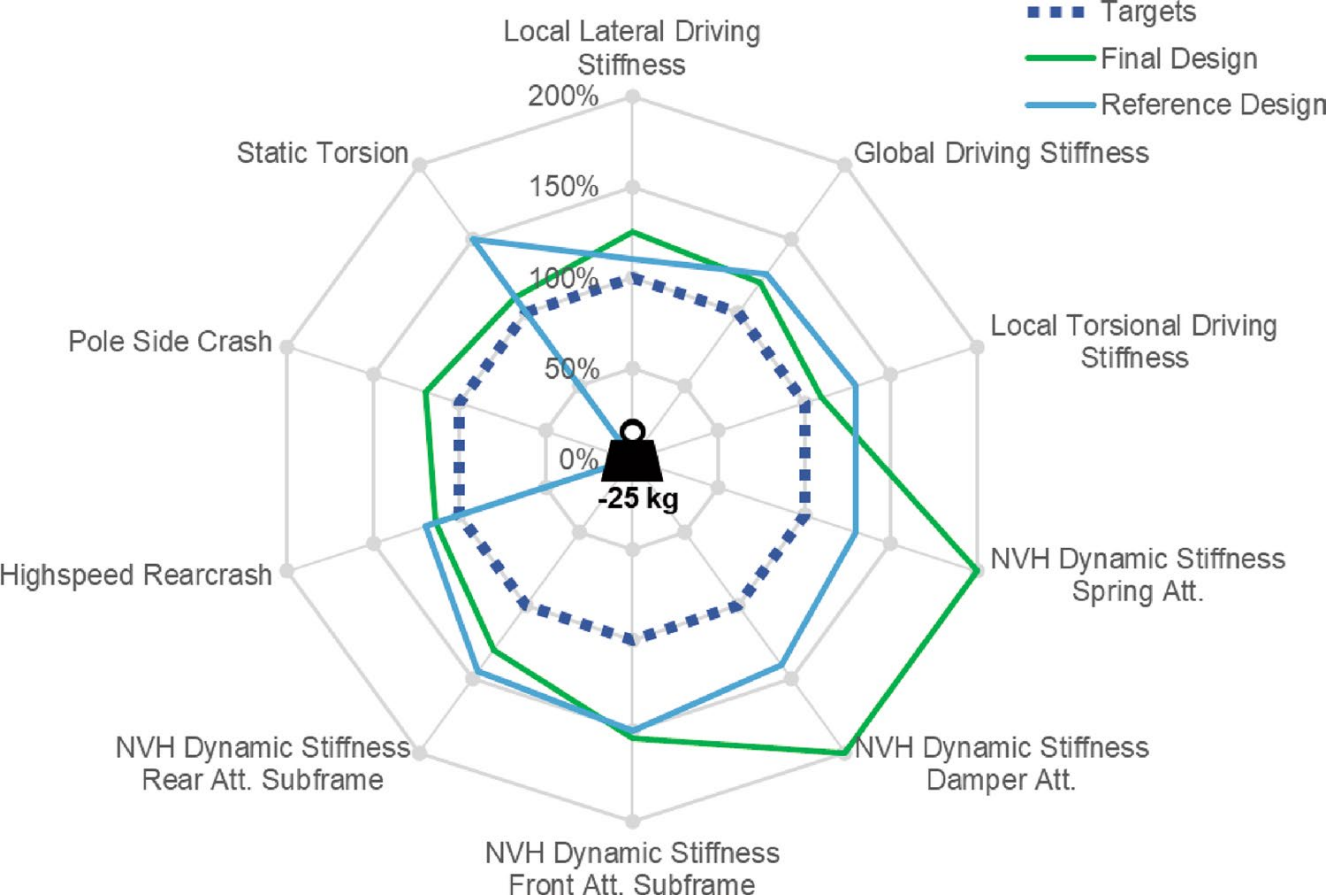
Performance of design resulting of two-phase generative design approach (final design) compared to design stemming from classical design process (reference design).

The previously described two-phase design approach yields the design illustrated in Fig. 24. In the following, this design is validated by comparison with a design stemming from classical design process. Figure 25 compares the performance of both with respect to summarized quantified design requirements. As can be seen, for most requirements both designs overperform. For the pole-side impact, however, requirements are not fulfilled by the reference design. In contrast to the two-phase design approach, the classical approach suffers from inefficient load paths and even more at identifying the structural integer failure modes with appropriate energy absorption.

The final design’s superiority becomes even more pronounced when comparing weight. The casting components mass is 25 kg less than the reference. This also validates the success of the first phase optimal load-path conceptual design.

## Summary

This paper presents a multidisciplinary design optimization process to generate light-weight structural casting parts fulfilling various structural requirements. These requirements stem from different automotive design disciplines and are all handled by various types of finite element simulations of diverse complexity and nonlinearity. On top of these design disciplines, the manufacturing of mega-castings adds further challenges to design the casting process but also the part design.

A two-phase approach was presented to design such geometries. The process consists of a first phase where the conceptual design is generated by applying topology optimization to multidisciplinary load-cases. Part requirements from structural performance and casting process were linearized or adapted to be implemented into a multi-load case topology and free-sizing optimization problem, coupled by a design space consisting of more than 6 million design variables. Structural requirements based on linearization techniques summed up to about 120 load cases and casting requirements were considered employing manufacturing constraints. Balancing the driving load cases yielded several optimal load-path conceptual designs. A best candidate design was selected and then transferred into a parameterized FE-model of base surface and rib design for structural statics and dynamics, explicit crash analyses and casting simulations.

This model served for RSM-based optimization, during the second phase, to minimize part weight and simultaneously fulfill all given performance requirements. For pole-side impact clustering constraint were employed to target a desired failure mode for several concurrent pole positions. This structurally integer failure mode is key to balance energy absorption and intrusion and to successfully apply RSM-based optimization. Moreover, the ingate design was optimized considering both castability and crash performance for pole-side impact. The primary findings of this process underscore the pivotal role of ingate positioning in attaining a uniform flow distribution for balanced filling. Furthermore, optimizing the ingate position provides the opportunity to reduce the number of ingates to 8 while maintaining a filling time equivalent to using 10 ingates, resulting in cost savings on materials and enhancing both the castability and performance of the part by enhancing material quality on the most critical structural region.

Finally, the resulting structure has been validated by means of a comparison with a design stemming from classical design approach. The generative design result outperforms the reference in multiple ways. First, a weight reduction of 25 kg could be achieved. Second, requirements with regard to pole-side impact could only be fulfilled by the generative design result. To the authors' knowledge, no comparable optimization methodology has yet been published, considering multidisciplinary.

## Discussion

The presented procedure is in line with the current trend to leverage more detailed and brought simulation and—where data is available—usage of data analytics and machine learning. It involves heavy computational efforts, running up to 600 samples during DOE and optimum validation. Each crash simulation was run on a high-performance cluster with up to 128 CPU and sometimes more than 12 h of computing time.

The benefit of this massive algorithmic effort is twofold. First aspect is gained from an efficiency standpoint of the final product in terms of weight and passive crash safety when compared to the design stemming from classical design approach. Also, from a time-to-market perspective the efficiency gain is significant, as it converges rather quick to a weight optimal geometry. Involved heavy computation during the second phase RSM-based optimizations is mostly in parallel, because most runs are conducted during the initial sampling in the DOE-step. This allows a turnaround time of several weeks which is highly competitive in typical automotive design cycles.

The second type of benefit of the proposed generative design approach refers to its effectiveness compared to the classical design approach. This is reflected by the enabling aspect based on two challenges. One challenge is the high level of expertise necessary to successfully design such large casting parts. The presented machine-learning based enhancement for RSM-based optimization allows intuitive analysis and constraint definition of complex, simulation-based behavior of the design in crash or casting simulations. Thus, at the cost of significantly more simulation runs also less skilled experts can define appropriate performance constraints. Where usually only a small number of experts in large OEMs can contribute to such design projects, the generative design approach allows to scale by enabling enough computing power but less skilled personnel.

Finally, the second enabling challenge also addressed by the proposed generative design approach is the immense design freedom that HPDC manufacturing allows. For manufacturing of casted parts, it does hardly matter whether a surface has constant or varying thickness, whether rib patterns are simple and rectangular or arbitrarily shaped as long as distances and min/max thicknesses are fulfilled. Such a tremendous design freedom is prone to be handled successfully only by heavy usage of algorithms and computing power. The usual iterative design process based on CAD geometry and simulation analysis is not capable of exploring the full design freedom of a casting part, therefore missing the weight optimal design.

From an accuracy point of view, both compared approaches perform equally well. Similar FE-models are used in the final design phase leading to established accuracy in automotive design.

On top of the final weight-optimal design and comparably small turnaround time the applied algorithms deliver valuable information stemming from data analytics and sensitivity information at no additional cost. This allows for instance a sensitivity analysis comparing and ranking different structural requirements. Individual load-cases can be evaluated with respect to their contribution to the final part weight. Design space limitations or manufacturing concepts like draw direction sectioning and constraints like upper and lower thickness limits can be quantified and ranked. This allows to quantify further, higher ranking boundary conditions like manufacturing cost.

## Outlook

As outlook we refer to some important methodical improvements for the individual steps. Looking on the first phase, the linearization of nonlinear structural requirements can be improved or automatized by new developments like the DiESL method^48,50, 64^, providing a well-defined procedure to linearize crash load cases. The adoption of this methodology would involve several advantages: first, nonlinearities in geometry and material—prominent in crash—would find consideration in the linear auxiliary load cases, resulting in an improved approximation of the actual crash load cases. Second, time-consuming pre-processing steps, e.g. finding adequate modelling of impactors, determining trigger loads or load balancing would be reduced and replaced by automized procedure.

Moreover, more sophisticate manufacturing constraints could be introduced in phase one, e.g. maximum/minimum member size depending on the distance to the inlet, to enhance castability.

Phase-two is mostly related to RSM-based multidisciplinary optimization, which represent a very active field of research. Focusing on car crash as domain we refer to^65^ for a very recent summary of improvements for practical application in BIW design. We just mention so-called “Auto-ML” methods in the field of machine learning to give an idea on the large field of research regarding metamodeling as such. As proposed above the inclusion of classification into metamodeling for crash is quite new and further research to efficiently integrate this into typical regression-based optimization is necessary. Regarding the optimization itself based on metamodels, the selection and hyperparameter tuning and optimization represent current research activity as well. Particularly in the domain of car crash, we refer to^66,67^ for recent developments. Including further machine learning based prediction into a multifidelity type of optimization is a promising and active field of research. Data-driven prediction methods are rising to disrupt long simulations times or tedious repetitive simulation cycles. The overall target of all these activities is to improve the automatization within phase-two and to tackle the still large amount of computing resources to solve individual crash simulations during optimization, limiting the dimensionality of design space. In addition to the challenge of reducing CPU times directly, there are other challenges that arise during the implementation of the methodology: first the preparation of such MDO includes some organizational challenges. Those are for example the selection of relevant disciplines and related requirements to be included into the MDO. Moreover, the number of design variables and most relevant parts must be selected. Both decisions can have significant influence on turnaround time, optimization outcome, and involved CPU time and should therefore be done profoundly. Furthermore, technical challenges to be considered are the necessity of mapping design variables between different models, e.g. casting and crash models. Depending on the type of design variable, e.g. sizing or shape, the related complexity increases and future research could support further improvement of MDO.

In conclusion, the presented two-phase multidisciplinary optimization process to generate automotive mega-casting parts is an industry-ready solution. We believe that such solutions will spread significantly towards other examples as the convergence of simulation and data-driven methods will grow. The combination of gradient-based optimization for large number of design variables and RSM-based optimization for nonlinear and complex structural problems including manufacturing is applicable in a broad range of areas, e.g. aerospace, heavy industry, consumer electronics, etc. Generating large datasets by simulation and at the same time exploring the same large datasets by big data analytics and machine learning offers tremendous potential in all those areas. Capabilities of data storage and exploration in the cloud enable dedicated resource and development time management leveraging historical data. Clustering, classification and physics prediction are recently published in commercial software tools, e.g. ALTAIR ExpertAI, ALTAIR PhysicsAI.

## Data Availability

The data that support the findings of this study are available from BMW Group but restrictions apply to the availability of these data, which were used under license for the current study, and so are not publicly available. Data are however available from the authors upon reasonable request and with permission of BMW Group.

## References

[1] Mercedes-Benz. BIONEQXXTM casting. https://group-media.mercedes-benz.com/marsMediaSite/en/instance/picture/Mercedes-Benz-VISION-EQXX.xhtml?oid=52282796 (2022).

[2] Volvo Cars. Volvo Cars to invest SEK 10bn in Torslanda plant for next generation fully electric car production. https://www.media.volvocars.com/global/en-gb/media/pressreleases/294360/volvo-cars-to-invest-sek-10bn-in-torslanda-plant-for-next-generation-fully-electric-car-production-1 (2022).

[3] Kallas, K. M. Multi-directional unibody casting machine for a vehicle frame and associated methods (2018).

[4] Carney, D. Tesla’s Switch to Giga Press Die Castings for Model 3 Eliminates 370 Parts Article-Tesla’s Switch to Giga Press Die Castings for Model 3 Eliminates 370 Parts. Tesla’s Switch to Giga Press Die Castings for Model 3 Eliminates 370 Parts Article-Tesla’s Switch to Giga Press Die Castings for Model 3 Eliminates 370 Parts.

[5] Lehmhus, D. Advances in metal casting technology: A review of state of the art, challenges and trends—Part I: changing markets, changing products. *Metals* **12**. 10.3390/met12111959 (2022).

[6] Wärmefjord, K., Hansen, J. & Söderberg, R. Challenges in geometry assurance of megacasting in the automotive industry. *J. Comput. Inf. Sci. Eng.* **23** (2023).

[7] Rai, A. *et al.* Integrated energy absorbing castings. *Patent Publication Number WO/2022/031991* **5**, (2021).

[8] Ollar, J. *A Multidisciplinary Design Optimisation Framework for Structural Problems with Disparate Variable Dependence*. (2016).

[9] Duddeck F (2008). Multidisciplinary optimization of car bodies. Struct. Multidiscip. Optim..

[10] Wang W, Gao F, Cheng Y, Lin C (2017). Multidisciplinary design optimization for front structure of an electric car body-in-white based on improved collaborative optimization method. Int. J. Autom. Technol..

[11] Büttner, J., Schumacher, A., Bäck, T., Schwarz, S. & Krause, P. Making multidisciplinary optimization fit for practical usage in car body development. *Struct. Multidiscip. Optim.* **66**, (2023).

[12] Cramer, E. J., Dennis, Jr., J. E., Frank, P. D., Lewis, R. M. & Shubin, G. R. Problem formulation for multidisciplinary optimization. *SIAM J. Optim.* **4**, (1994).

[13] Sobieszczanski-Sobieski, J. & Haftka, R. T. Multidisciplinary aerospace design optimization: Survey of recent developments. In *34th Aerospace Sciences Meeting and Exhibit*. 10.2514/6.1996-711 (1996).

[14] Martins, J. R. R. A. & Lambe, A. B. Multidisciplinary design optimization: A survey of architectures. *AIAA J.* **51**, (2013).

[15] Bendsøe MP, Kikuchi N (1988). Generating optimal topologies in structural design using a homogenization method. Comput. Methods Appl. Mech. Eng..

[16] Sigmund, O. & Maute, K. Topology optimization approaches: A comparative review. *Struct. Multidiscip. Optim*. **48**, 1031–1055. 10.1007/s00158-013-0978-6 (2013).

[17] Altair Engineering Inc. *OptiStruct 2022.3 User’s Guide* (Altair Engineering, Inc., 2021).

[18] Bendsøe MP (1989). Optimal shape design as a material distribution problem. Struct. Optim..

[19] Zhou M, Rozvany GIN (1991). The COC algorithm, Part II: Topological, geometrical and generalized shape optimization. Comput. Methods Appl. Mech. Eng..

[20] Mlejnek HP (1992). Some aspects of the genesis of structures. Struct. Optim..

[21] Haftka RT, Gürdal Z, Kamat MP (2012). Elements of structural optimization.

[22] Kicinger R, Arciszewski T, De Jong K (2005). Evolutionary computation and structural design: A survey of the state-of-the-art. Comput. Struct..

[23] Rechenberg, I. *Evolutionsstrategie’94*. (Friedrich Frommann Verlag, 1994).

[24] Rechenberg I (1970). Optimierung technischer Systeme nach Prinzipien der biologischen Evolution.

[25] Hornik, K., Stinchcombe, M. & White, H. Universal approximation of an unknown mapping and its derivatives. Artificial Neural Networks: Approximations and Learning Theory. *Neural Netw.* **3**, 551–560.

[26] Hornik, K., Stinchcombe, M. & White, H. Universal approximation of an unknown mapping and its derivatives. In *Neural networks: Approximation and Learning Theory* (Blackwell, 1992).

[27] Cressie N (1990). The origins of kriging. Math. Geol..

[28] Cressie N (1988). Spatial prediction and ordinary kriging. Math. Geol..

[29] Matheron G (1963). Principles of geostatistics. Econ. Geol..

[30] Krige, D. G. A statistical approach to some basic mine valuation problems on the Witwatersrand (University of the Witwatersrand, 1951).

[31] Hardy RL (1971). Multiquadric equations of topography and other irregular surfaces. J. Geophys. Res..

[32] Fang H, Rais-Rohani M, Liu Z, Horstemeyer MF (2005). A comparative study of metamodeling methods for multiobjective crashworthiness optimization. Comput. Struct..

[33] Fang J, Sun G, Qiu N, Kim NH, Li Q (2017). On design optimization for structural crashworthiness and its state of the art. Struct. Multidiscip. Optim..

[34] Ortmann C, Schumacher A (2013). Graph and heuristic based topology optimization of crash loaded structures. Struct. Multidiscip. Optim..

[35] Beyer F, Schneider D, Schumacher A (2020). Finding three-dimensional layouts for crashworthiness load cases using the graph and heuristic based topology optimization. Struct. Multidiscip. Optim..

[36] Patel, N. M. Crashworthiness design using topology optimization (University of Notre Dame, 2007).

[37] Patel, N. M., Kang, B.-S., Renaud, J. E. & Tovar, A. Crashworthiness design using topology optimization. *J. Mech. Des.* **131**, (2009).

[38] Von Neumann, J. The general and logical theory of automata, cerebral mechanisms in behavior. The Hixon Symposium (Wiley, New York, 1951).

[39] Park GJ (2011). Technical overview of the equivalent static loads method for non-linear static response structural optimization. Struct. Multidiscip. Optim..

[40] Shin MK, Park KJ, Park GJ (2007). Optimization of structures with nonlinear behavior using equivalent loads. Comput. Methods Appl. Mech. Eng.

[41] Park KJ, Lee JN, Park GJ (2005). Structural shape optimization using equivalent static loads transformed from dynamic loads. Int. J. Numer. Methods Eng..

[42] Kim, Y., & Park, G. J. Nonlinear dynamic response structural optimization using equivalent static loads. *Comput. Methods Appl. Mech. Eng.* **199**, 660–676 (2010).

[43] Choi WS, Park GJ (2002). Structural optimization using equivalent static loads at all time intervals. Comput. Methods Appl. Mech. Eng..

[44] Lee Y, Ahn J-S, Park G-J (2015). Crash optimization of an automobile frontal structure using equivalent static loads. Trans. Korean Soc. Autom. Eng..

[45] Choi WH, Lee Y, Yoon JM, Han YH, Park GJ (2018). Structural optimization for roof crush test using an enforced displacement method. Int. J. Autom. Technol..

[46] Jeong, S. B., Yi, S. I., Kan, C. D., Nagabhushana, V. & Park, G. J. Structural optimization of an automobile roof structure using equivalent static loads. *Proc. Inst. Mech. Eng. Part D J. Autom. Eng.*10.1243/09544070JAUTO855 (2008).

[47] Karev A, Harzheim L, Immel R, Erzgräber M (2019). Free sizing optimization of a front hood using the ESL method: Overcoming challenges and traps. Struct. Multidiscip. Optim..

[48] Triller J, Immel R, Timmer A, Harzheim L (2021). The difference-based equivalent static load method: An improvement of the ESL method’s nonlinear approximation quality. Struct. Multidiscip. Optim..

[49] Stolpe M, Verbart A, Rojas-Labanda S (2018). The equivalent static loads method for structural optimization does not in general generate optimal designs. Struct. Multidiscip. Optim..

[50] Triller, J., Immel, R. & Harzheim, L. Topology optimization using difference—based equivalent static loads. *Struct. Multidiscip. Optim.* **7**, (2022).

[51] Duddeck, F. & Volz, K.-H. A new topology optimization approach for crashworthiness of passenger vehicles based on physically defined equivalent static loads. *ICRASH2012* (2012).

[52] Clemens P, Schumacher A (2023). Nested loop approach for topology and shape optimization of crash-loaded deep-drawn components using contact forces for the inner loops. Structures.

[53] Ollar, J. Systems and methods for improving a design of article using expert emulation. (2022).

[54] Zoutendijk, G. Methods of feasible directions. A study in linear and non-linear programming. Preprint at (1960).

[55] Powell, M. J. D. The theory of radial basis function approximation in 1990, Advances in Numerical Analysis II: Wavelets, Subdivision, and Radial Functions (WA Light, ed.). *Oxford University Press, Oxford* **105**, 105–210 (1992).

[56] Barthelemy J-FM, Haftka RT (1993). Approximation concepts for optimum structural design—a review. Struct. Optim..

[57] Wang GG, Shan S (2007). Review of metamodeling techniques in support of engineering design optimization. J. Mech. Des..

[58] Forrester AIJ, Keane AJ (2009). Recent advances in surrogate-based optimization. Prog. Aerosp. Sci..

[59] Viana, F. A. C., Gogu, C. & Haftka, R. T. Making the most out of surrogate models: tricks of the trade. In *ASME 2010 International Design Engineering Technical Conferences and Computers and Information in Engineering Conference* 587–598 (2010).

[60] Viana FAC, Simpson TW, Balabanov V, Toropov V (2014). Metamodeling in multidisciplinary design optimization: How far have we really come?. Prog. Aerosp. Sci..

[61] Altair Engineering, Inc. HyperStudy Simulation Help. Preprint at (2023).

[62] NADCA. Gating Manual. Preprint at (2006).

[63] Lopez, M. Guidelines to product simulation-driven-design optimization—topology and casting application. (Universidad Politecnica De Madrid, 2021).

[64] Triller J, Immel R, Harzheim L (2022). Difference-based equivalent static load method with adaptive time selection and local stiffness adaption. Struct. Multidiscip. Optim..

[65] Büttner J, Schumacher A, Bäck T, Schwarz S, Krause P (2023). Making multidisciplinary optimization fit for practical usage in car body development. Struct. Multidiscip. Optim..

[66] Long, F. X. *et al.* Towards surrogate-based automated algorithm selection and hyperparameter tuning. In *submitted to ACM Transactions on Evolutionary Computation and Learning (ACM TELO)—special issue ‘Best of GECCO 2022’* (2023). 10.1145/3512290.3528712.

[67] Long, F. X. *et al.* Learning the characteristics of engineering optimization problems with applications in automotive crash. In *GECCO 2022—Proceedings of the 2022 Genetic and Evolutionary Computation Conference* (2022). 10.1145/3512290.3528712.

